# Tomato SlPUB24 enhances resistance to *Xanthomonas euvesicatoria* pv. *perforans* race T3

**DOI:** 10.1038/s41438-021-00468-4

**Published:** 2021-02-01

**Authors:** Xin Liu, Ge Meng, Mengrui Wang, Zilin Qian, Yaxian Zhang, Wencai Yang

**Affiliations:** 1 Beijing Key Laboratory of Growth and Developmental Regulation for Protected Vegetable Crops, Department of Vegetable Science, China Agricultural University, Beijing, 100193 China; 2 Joint Laboratory for International Cooperation in Crop Molecular Breeding, Ministry of Education of the People’s Republic of China, Beijing, 100193 China

**Keywords:** Plant molecular biology, Biotic

## Abstract

*Solanum lycopersicum* var. *cerasiforme* accession PI 114490 has broad-spectrum resistance to bacterial spot caused by several species of *Xanthomonas*. Resistance is quantitatively inherited, and a common quantitative trait locus *QTL-11B* on chromosome 11 has been identified previously. In this study, the *SlPub24* gene was characterized in *QTL-11B*. *SlPub24* in PI 114490 was upregulated by infection with *X*. *euvesicatoria* pv. *perforans* race T3, but its transcription was low in the susceptible line OH 88119 whether or not it was infected by the pathogen. The differential expression of *SlPub24* between PI 114490 and OH 88119 was due to great sequence variation in the promoter region. The promoter of *SlPub24* in OH 88119 had very low activity and did not respond to pathogen infection. Transgenic lines of OH 88119 overexpressing *SlPub24* isolated from PI 114490 showed significantly enhanced resistance, while mutants of *Slpub24* generated by CRISPR/Cas9 editing showed more susceptibility to race T3 and to other races. The mutants also showed spontaneous cell death in leaves. The expression of the salicylic acid (SA) pathway gene phenylalanine ammonia-lyase (*PAL*) and signaling-related genes pathogenesis-related (*PR1)* and nonexpresser of *PR*1 (*NPR1*) were influenced by *SlPub24*. The content of SA in tomato plants was consistent with the level of *SlPub24* expression. Furthermore, SlPUB24 interacted with the cell wall protein SlCWP and could regulate the degradation of SlCWP. The expression levels of *SlCWP* and *SlCWINV1*, a cell wall invertase gene, showed opposite patterns during pathogen infection. The activity of SlCWINV1 was lower in mutants than in PI 114490. The results are discussed in terms of the roles of the abovementioned genes, and a potential model for SlPUB24-mediated resistance to bacterial spot is proposed.

## Introduction

Bacterial spot caused by *Xanthomonas euvesicatoria* pv. *euvesicatoria* (race T1), *X*. *vesicatoria* (race T2), *X*. *euvesicatoria* pv. *perforans* (races T3 and T4), and *X. cynarae* pv. *gardneri* is a widespread disease in tomato production^1–3^. The disease can cause severe yield loss and fruit quality reduction in tomato^4,5^. Although the use of resistant varieties is the most effective approach for control of the disease, the existence of multiple species of *Xanthomonas* and quick shifts of species/races in the same region are among the most important causes of unsuccessful management of the disease^4–6^. Therefore, sources with more durable and broad-spectrum resistance to the disease are desirable for developing new cultivars.

Several studies have indicated that *Solanum lycopersicum* var. *cerasiforme* accession PI 114490 may provide broad-spectrum resistance to all species and races of *Xanthomonas* causing bacterial spot in tomato^7–9^. The resistance to races T1–T4 and *X. cynarae* pv. *gardneri* in PI 114490 is quantitatively inherited, and several quantitative trait loci (QTLs) have been reported^7–13^. Classical genetic analyses based on segregation of resistance in F_2_ and inbred backcross (IBC) populations derived from PI 114490 suggest that its resistance to race T2 is conditioned by two to four loci^8^. The high correlation between race T1 and race T2 resistance in the IBC population suggests that there are common loci for resistance to both races, while the poor correlation of resistance between races T2 and T3 indicates that resistance to all species and races is not controlled by the same genes in PI 114490^8^. However, a common locus on chromosome 11 conferring resistance to races T2, T3, and T4 has been identified in the same IBC population^13^. The common locus conferring resistance to races T3 and T4 has also been confirmed in later studies using the same IBC population and other segregating populations^10,12^. Recent studies indicate that the common locus on chromosome 11, designated *QTL-11B*, confers resistance to races T1–T4 and *X. cynarae* pv. *gardneri*^7,9^. All these data suggest that *QTL-11B* in PI 114490 confers resistance to all races and species. Previous studies have shown that the *Solyc11g068940* gene, encoding a plant U-box protein (designated SlPUB24 in this study), in the *QTL-11B* region of PI 114490 is highly induced by the presence of the race T3 strain^14,15^, suggesting that it may participate in resistance to race T3.

E3 ubiquitin (Ub) ligases are key regulators in plants for defense during both PAMP-triggered immunity (PTI) and effector-triggered immunity (ETI). Their regulation can be either positive or negative depending on the plant-pathogen system and usually involves the production of hydrogen peroxide (H_2_O_2_) and salicylic acid (SA). SPL11 in rice, the first characterized U-box E3 ligase, acts as a negative regulator of plant programmed cell death (PCD) and pathogenic defense. The *spl11* mutant shows spontaneous cell death in leaves and confers enhanced resistance to rice blast and bacterial blight^16,17^. PUB13, the closest ortholog of SPL11 in *Arabidopsis*, negatively regulates resistance to the biotrophic pathogens *Pseudomonas syringae* pv. *maculicola* and *Erysiphe cichoracearum* but positively regulates resistance to the necrotrophic fungal pathogens *Botrytis cinerea* and *Alternaria brassicicola*. The spontaneous cell death and elevated H_2_O_2_ accumulation in the *pub13* mutant depend on the SA signal^18,19^. PUB22, PUB23, and PUB24 in *Arabidopsis* act as negative regulators of PTI in response to several distinct PAMPs. The triple mutant *pub22/pub23/pub24* exhibits enhanced resistance to diverse pathogens, accompanied by oxidative burst and plant cell death^20,21^. PUB17, a U-box ARM repeat E3 ligase conserved in *Arabidopsis*, *Nicotiana benthamiana*, tomato and potato^22,23^, is a positive regulator of cell death and plant disease resistance. Another conserved class of U-box E3 ligases, including CMPG1 in *Petroselinum crispum*^24^, PUB20 and PUB21 in *A. thaliana*^25^, CMPG1-V in *Haynaldia villosa*^26^, NtCMPG1 in *N. tabacum*, and SlCmpg1 in tomato^27^, also act as positive regulators of plant disease resistance. The pepper E3 ubiquitin ligase CaRING1 is a positive regulator of resistance and is required for cell death and the SA-dependent defense response to hemibiotrophic *Pseudomonas syringae* pv. *tomato* and biotrophic *Hyaloperonospora arabidopsidis* infections^28^. In apple fruits, two ubiquitin E3 ligases regulate the immune response with opposing functions. The U-box E3 ligase MdPUB29 is a positive regulator of the defense response to the fungal pathogen *Botryosphaeria dothidea*, possibly regulating the SA pathway^29^, while the BTB-BACK domain E3 ligase MdPOB1 ubiquitinates and degrades MdPUB29, resulting in suppression of defense against *B. dothidea*^30^.

Here, we reported that U-box E3 ligase protein 24 (SlPUB24) acted as a positive regulator of resistance to bacterial spot in tomato. The knockout mutants also exhibited spontaneous cell death in leaves. The increase in plant pathogen defense was correlated with the SA biosynthesis pathway and signaling. In addition, SlPUB24 targeted the potential cell wall protein SlCWP. A model is proposed to provide additional understanding of the U-box-mediated response to disease.

## Results

### Sequence variation in *SlPub24* between PI 114490 and OH 88119

**Fig. 1:**
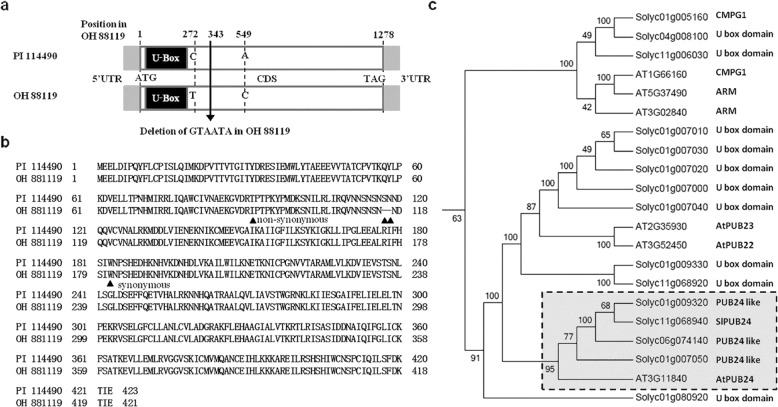
Comparison of *SlPub24* sequences between the resistant line PI 114490 and the susceptible line OH 88119, as well as phylogenetic analysis of SlPUB24. **a** Diagram shows differences in coding sequences of *SlPub24* between PI 114490 and OH 88119. **b** Alignment of deduced amino acid sequences of SlPUB24 between PI 114490 and OH 88119. ▲ indicates the amino acid substitution or insertion/deletion positions. **c** Phylogenetic analysis of SlPUB24 with various U-box type E3 ubiquitin ligases in *Solanum lycopersicum* and *Arabidopsis thaliana*

The genomic DNA sequence of the *SlPub24* gene was obtained by PCR amplification using gene-specific primers (Table S1). It was 1278 bp in the resistant line PI 114490 and 1272 bp in the susceptible line OH 88119. Full-length cDNA was obtained by RT-PCR and RACE. A 1457-bp fragment and a 1451-bp fragment were obtained for PI 114490 and OH 88119, respectively. Alignment of genomic DNA and cDNA sequences revealed that there was no intron in the gene. There were two single nucleotide polymorphisms (SNPs) and one 6-bp (GTAATA) insertion/deletion (InDel) in the coding region (Fig. 1a) and no sequence variation in the 5′UTR (86 bp) or 3′UTR (93 bp) between PI 114490 and OH 88119. A comparison of deduced amino acid sequences showed that the 6-bp InDel resulted in two amino acid losses in OH 88119, while C272T was a nonsynonymous substitution (T91I), and A549C was a synonymous substitution (Fig. 1b). A U-box domain at the N-terminus of the deduced protein sequence between amino acids 9 and 79 (25–237 bp in cDNA sequence, Fig. 1a) was predicted with SMART (http://smart.embl-heidelberg.de/). Phylogenetic analysis indicated that the deduced protein was closely related to AtPUB24 in *Arabidopsis* (Fig. 1c), which confirmed the nomenclature of SlPUB24. A BLAST search of SlPUB24 in the tomato genome ITAG release 4.0 (https://solgenomics.net/) identified four genes annotated as U-box domain-containing protein 24 (Fig. 1c), but their coding sequences were quite different (Fig. S1).

### SlPUB24 is a ubiquitous protein

**Fig. 2:**
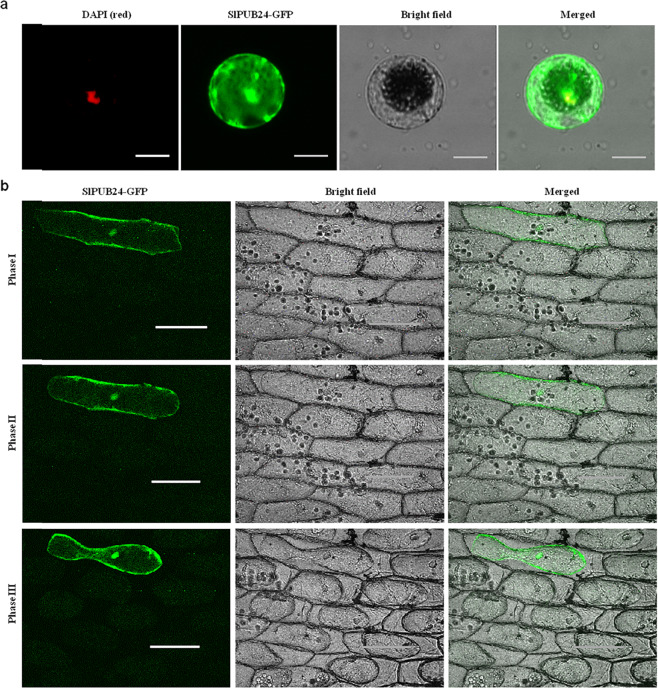
Subcellular localization of SlPUB24. **a** Subcellular localization of SlPUB24-GFP in tomato protoplasts isolated from tomato line PI 114490. **b** Dynamic observation of subcellular localization of SlPUB24-GFP in onion epidermal cells infiltrated with 0.3g/ml sucrose solution. Scale bars = 200 μm

The subcellular localization of the SlPUB24 protein was determined by *Agrobacterium*-mediated transient expression of the *SlPub24* gene in tomato protoplast and onion epidermal cells. The SlPUB24-GFP fusion protein was detected in the cytoplasm, plasma membrane, and nucleus in tomato cells (Fig. 2a), and this localization was confirmed by observation of the SlPUB24-GFP fusion protein during plasmolysis of onion epidermal cells (Fig. 2b). These data suggested that SlPUB24 is a ubiquitous protein.

### Transgenic overexpression of *SlPub24* in OH 88119 enhances resistance to *X. euvesicatoria* pv. *perforans* race T3

**Fig. 3:**
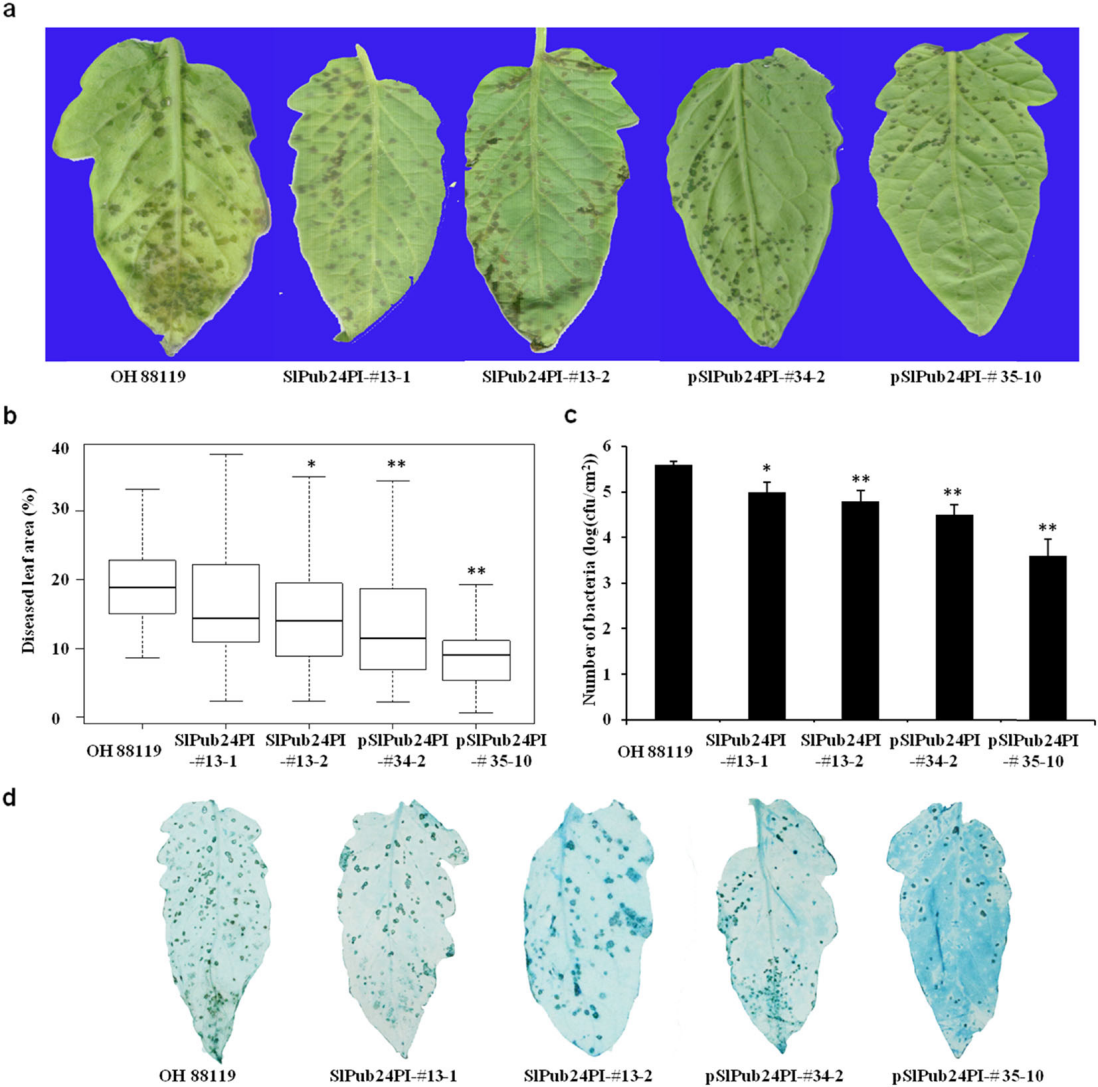
Transgenic overexpression of *SlPub24* in the susceptible tomato line OH 88119 enhances resistance to *Xanthomonas euvesicatoria* pv. *perforans* race T3 strain *Xv829*. **a** Symptoms in leaves of OH 88119 and transgenic plants overexpressing *SlPub24* isolated from PI 114490 at 7 days post inoculation (dpi). **b** Statistical analysis of disease in OH 88119 and transgenic lines at 7 dpi. Error bars represent the SD (*n* = 30). **c** Bacterial populations in leaves of OH 88119 and transgenic lines at 9 dpi. Error bars represent the SD (*n* = 30). **d** Trypan blue staining of diseased leaves of OH 88119 and transgenic lines (*n* = 30). The asterisks indicate statistical significance by *t* test (*P < 0.05, **P < 0.01)

To determine the role of the *SlPub24* gene in PI 114490 in resistance to *X. euvesicatoria* pv. *perforans* race T3 strain *Xv829*, two constructs, SlPub24PI (Fig. S2a) and pSlPub24PI (Fig. S2b), were developed for overexpression of the gene in the susceptible tomato line OH 88119. A total of 12 independent transgenic lines for the construct SlPub24PI and 4 independent transgenic lines for the construct pSlPub24PI were obtained. Four transgenic lines, SlPub24PI-#13-1, SlPub24PI-#13-2, pSlPUB24PI-#34-2, and pSlPub24PI-#35-10, were used in the following experiments. All transgenic plants had fewer disease lesions on leaves than OH 88119 plants at 9 days post inoculation (dpi) (Fig. 3a). The percentage of diseased leaf area was significantly (*P* < 0.05) lower in the three transgenic lines SlPub24PI-#13-2, pSlPub24PI-#34-2, and pSlPub24PI-#35-10 than in OH 88119 (Fig. 3b), and the bacterial populations were significantly (*P* < 0.05) smaller in all transgenic lines than in OH 88119 (Fig. 3c). The leaves of transgenic plants had less necrotic tissue than OH 88119 (Fig. 3d). The transgenic lines carrying the *SlPub24* gene with its native promoter from PI 114490 (construct pSlPub24PI) were more resistant than the transgenic lines carrying only *SlPub24* from PI 114490 (construct SlPub24PI), suggesting that the native promoter and UTRs might enhance the resistance.

### Mutation of *SlPub24* in PI 114490 by CRISPR/Cas9 editing increases susceptibility to *X. euvesicatoria* pv. *perforans* race T3 and induces spontaneous cell death in leaves

**Fig. 4:**
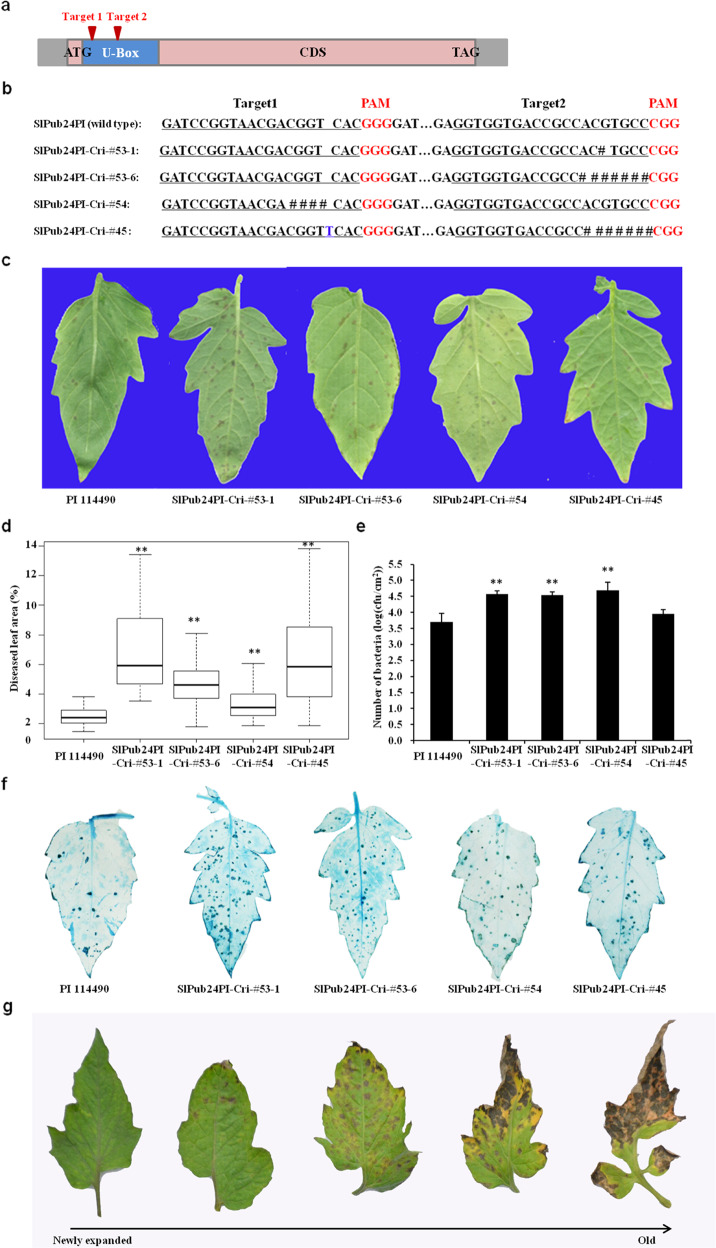
Knockout of *SlPub24* in the resistant line PI 114490 by the CRISPR/Cas9 editing system leads to decreased resistance to *Xanthomonaseuvesicatoria* pv. *perforans* race T3 strain *Xv829* and spontaneous cell death in leaves in mutants. **a** Schematic illustration of the two sgRNA target sites (red arrows) in *SlPub24*. **b** Mutations identified in four T2 mutant lines. Red font indicates protospacer-adjacent motif (PAM) sequences, and the sgRNA target sequence is underlined. **c** Symptoms on PI 114490 and mutant leaves at 7 days post inoculation (dpi). **d** Statistical analysis of disease in PI 114490 and mutants at 7 dpi. Error bars represent the SD (*n* = 30). **e** Bacterial population in leaves of PI 114490 and mutants at 9 dpi. Error bars represent the SD (*n* = 30). **f** Trypan blue staining of diseased PI 114490 and mutant leaves (*n* = 30). The asterisks indicate statistical significance by *t* test (**P < 0.01). **g** Spontaneous cell death on leaves of mutants at various stages, from newly expanded to old leaves

The *SlPub24* gene was mutated in PI 114490 at two target sites (Fig. 4a) using the CRISPR/Cas9 editing system to further validate the role of *SlPub24* in resistance to *X. euvesicatoria* pv. *perforans* race T3. Three mutated lines (SlPub24PI-Cri-#53, SlPub24PI-Cri-#54, and SlPub24PI-Cri-#45) were obtained. The line SlPub24PI-Cri-#53 had two types of mutations in the target 2 region in T1 progenies: a 1-bp deletion in line SlPub24PI-Cri-#53-1 and a 7-bp deletion in line SlPub24PI-Cri-#53-6. Line SlPub24PI-Cri-#54 had a 4-bp deletion in the target 1 region, and line SlPub24PI-Cri-#45 had a T insertion in the target 1 region and a 7-bp deletion in the target 2 region (Fig. 4b). Leaves of mutants showed more disease lesions than those of PI 114490 (Fig. 4c). The percentage of diseased leaf area (Fig. 4d) and bacterial populations in leaves (Fig. 4e) were significantly (*p* < 0.01) higher in mutants than in PI 114490 at 7 dpi and 9 dpi, respectively. Mutants had more necrotic tissue in leaves than did PI 114490 (Fig. 4f). These results indicated that *SlPub24* contributed to resistance to race T3.

Spontaneous cell death in leaves was also observed in *SlPub24*-mutated plants that had not been inoculated with *Xv829*. Newly expanded leaves were normal, but areas of cell death were observed several days after expansion (Fig. 4g), and the leaves eventually became completely withered.

### Differential expression of *SlPub24* in PI 114490 and OH 88119 during disease development is caused by promoter sequence variation

**Fig. 5:**
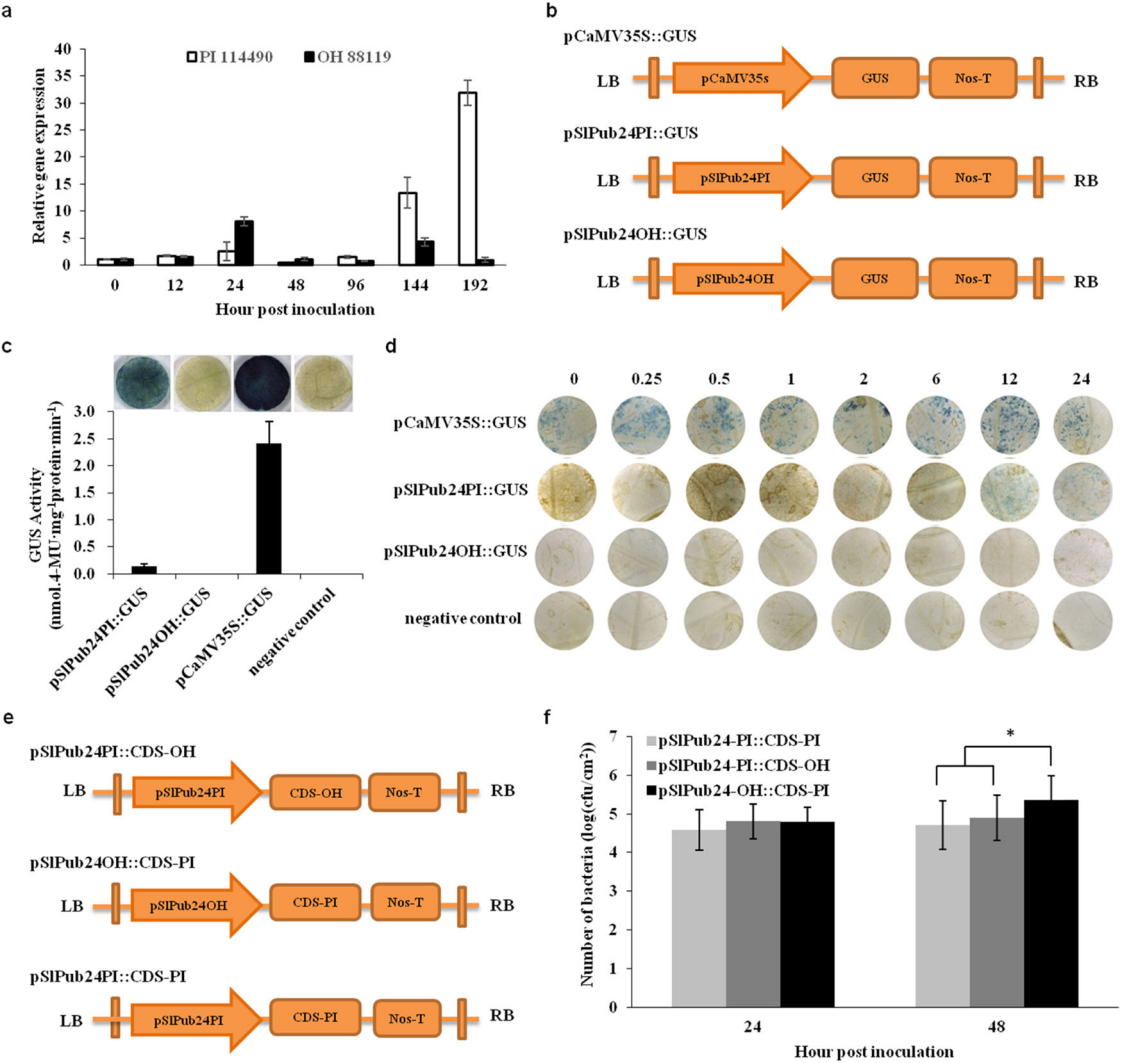
Differential expression of *SlPub24* in the resistant line PI 114490 and the susceptible line OH 88119 during infection by *Xanthomonas euvesicatoria* pv. *perforans* race T3 strain *Xv829* is caused by sequence variation in the promoter. **a** Expression of *SlPub24* in PI 114490 and OH 88119 at various time points after spray inoculation with *Xv829*. **b** Schematic diagram of the vector construct. pSlPub24PI::GUS: The *SlPub24* promoter isolated from PI 114490 was fused with the GUS reporter. pSlPub24OH::GUS: The *SlPub24* promoter isolated from OH 88119 was fused with the GUS reporter. GUS β-glucuronidase, LB left border, NOS-T Nos terminator, RB right border. *Agrobacterium tumefaciens* GV3101 was used as a negative control, and pCaMV35S::GUS was used as a positive control. **c** GUS activity in leaves of *Nicotiana benthamiana* transiently expressing the pSlPub24PI::GUS or pSlPub24OH::GUS constructs. Error bars represent the SD (*n* = 6). **d** GUS activity in tomato leaves transiently expressing the pSlPUB24OH::GUS and pSlPUB24PI::GUS constructs at various hours after spray inoculation of *Xv829*. *Agrobacterium strains* carrying the constructs were infiltrated into tomato leaves at 48 h before inoculation with *Xv829*. **e** Schematic diagram of the vector construct. SlPub24PI::CDS-PI: the *SlPup24* coding sequence (CDS) from PI 114490 driven by promoter from PI 114490; SlPub24PI::CDS-OH: the *SlPub24* CDS from OH 88119 driven by promoter from PI 114490; SlPub24OH::CDS-PI: the *SlPub24* CDS from PI 114490 driven by promoter from OH 88119. **f** Bacterial population in leaves of OH 88119 plants transiently expressing constructs SlPub24PI::CDS-PI, SlPub24PI::CDS-OH, and SlPub24OH::CDS-PI. Error bars represent the SD (*n* = 6). The asterisks indicate statistical significance by *t* test (*P < 0.05)

Differential expression of *SlPub24* was observed in PI 114490 and OH 88119 during infection by *X. euvesicatoria* pv. *perforans* race T3 strain *Xv829*. In PI 114490, the expression of *SlPub24* was at a constant low level from 0 to 96 hpi and then dramatically increased, which was consistent with previous findings^14,15^. However, the expression of *SlPub24* in OH 88119 remained at a low level from 0 to 192 hpi (Fig. 5a).

To investigate why the expression of *SlPub24* showed different patterns in PI 114490 and OH 88119, approximately 2.4 kb promoter sequences were obtained from the genomic DNA of these two lines. Substantial sequence variation in the promoter region of *SlPub24* was observed between PI 114490 and OH 88119. There were two large InDels, seven small InDels, and 16 SNPs between the two promoter sequences (Table S2). Both promoters were separately fused with GUS (Fig. 5b) to determine their activities. The GUS activity assay in tobacco showed that the promoter activity of *SlPub24* from OH 88119 was lower than that of *SlPub24* from PI 114490 (Fig. 5c). GUS activity of the construct driven by the promoter isolated from PI 114490 was detected in leaves of OH 88119 at 12 hpi with *Xv829*, but no GUS activity of the construct driven by the promoter isolated from OH 88119 was detected in leaves of PI 114490 (Fig. 5d). These results suggested that the sequence difference in the promoter region might cause a difference in the activities of the two promoters, resulting in different expression patterns of *SlPub24* in response to infection with *Xv829* in PI 114490 and OH 88119.

Further comparison of promoter activities was performed by switching the promoters and coding sequences (CDSs) of the *SlPub24* gene originating from PI 114490 and OH 88119. Three constructs, pSlPub24PI::CDS-OH, pSlPub24OH::CDS-PI, and pSlPub24PI::CDS-PI (Fig. 5e), were developed to perform transient transformation experiments. The number of bacteria was significantly (*P* < 0.05) lower when *SlPub24* was driven by the promoter from PI 114490 than when it was driven by the promoter from OH 88119 at 48 hpi (Fig. 5f). These results suggested that the *SlPub24* gene could confer resistance to *X*. *euvesicatoria* pv. *perforans* race T3 and that the differential expression of *SlPub24* during pathogen infection in PI 114490 and OH 88119 might be due to sequence variation in the *SlPub24* promoter region.

### *SlPub24* is associated with resistance to race T3 in the inbred backcross population derived from PI 114490 and other germplasms

The 6-bp InDel in the CDS between PI 114490 and OH 88119 was used as a marker (Table S1) to genotype individual lines of an inbred backcross (IBC) population derived from PI 114490^8,12^. The responses of each line in the IBC population to race T3 were the same as those in our previous publication^12^. Single marker-trait association analysis indicated that the marker was significantly (*p* = 0.0056) associated with resistance to race T3 in the population. The mean disease severity was 3.9 (1–12 scale)^13^ for lines carrying the PI 114490 allele and 6.4 for lines with the OH 9242 or Fla 7600 allele. The marker explained 12.3% of the phenotypic variation in resistance to race T3 in the population.

Of the 192 tomato lines^31^ genotyped with markers to detect *SlPub24* and *Rx4*, including PI 114490 and OH 88119, 4 had only the *SlPub24* gene, 3 had only the *Rx4* gene, and 11 had both the *SlPub24* and *Rx4* genes (Table 1). Interestingly, nine lines showed evidence of chromosomal crossover events in this region. Seven lines had the PI 114490 marker genotype in the CDS and 5′UTR of *SlPub24* but the OH 88119 marker genotype in the promoter region. Two near-isogenic lines (NILs) derived from Hawaii 7998, FG16-804 and FG16-813, had the same genotype as the donor. Line LA1269 had the OH 88119 marker genotype in the CDS but PI 114490 marker genotypes at the promoter and 5′UTR regions, while line LA1218 had the OH 88119 marker genotype in the CDS and 5′UTR but the PI 114490 marker genotype at the promoter (Table 1). Twenty-six lines, including those with either the *SlPub24* or *Rx4* gene, 9 without both genes, and those with chromosomal crossovers, were subjected to disease evaluation by spray inoculation of race T3 strain *Xv829*. Lines carrying both the *SlPub24* and *Rx4* genes exhibited the lowest diseased leaf area (9.3%), followed by lines carrying only *Rx4* (11.0%) from PI 128216 or *SlPub24* (13.3%) from PI 114490 (Table 1). Lines having chromosomal crossover without either the promoter or CDS from PI 114490 exhibited the same level of susceptibility as lines without both *SlPub24* and *Rx4* genes. These data suggested that the promoter of *SlPub24* from PI 114490 was critical for the function of *SlPub24* in disease resistance and confirmed the results of switching the promoters and CDS of the *SlPub24* gene originating from PI 114490 and OH 88119.

**Table 1:** Marker genotype and response to *Xanthomonas euvesicatoria* pv. *perforans* race T3 strain *Xv829* in tomato lines

Germplasm	Marker genotype				Mean diseased leaf area (%)
	*SlPub24*			*Rx4*	
	284-bp InDel in promoter	198-bp InDel in 5′-UTR	6-bp InDel in CDS	6-bp InDel in CDS	
Money maker	Slpub24Slpub24	Slpub24Slpub24	Slpub24Slpub24	rx4rx4	22.2a
Liger 87-05	Slpub24Slpub24	Slpub24Slpub24	Slpub24Slpub24	rx4rx4	
Zhongshu 6	Slpub24Slpub24	Slpub24Slpub24	Slpub24Slpub24	rx4rx4	
Hunt 100	Slpub24Slpub24	Slpub24Slpub24	Slpub24Slpub24	rx4rx4	
Ailsa Craig	Slpub24Slpub24	Slpub24Slpub24	Slpub24Slpub24	rx4rx4	
Heinz 1350	Slpub24Slpub24	Slpub24Slpub24	Slpub24Slpub24	rx4rx4	
Heinz 1706	Slpub24Slpub24	Slpub24Slpub24	Slpub24Slpub24	rx4rx4	
OH 88119	Slpub24Slpub24	Slpub24Slpub24	Slpub24Slpub24	rx4rx4	
Rio Grande	Slpub24Slpub24	Slpub24Slpub24	Slpub24Slpub24	rx4rx4	
LA 1218	SlPub24SlPub24	Slpub24Slpub24	Slpub24Slpub24	rx4rx4	not included
LA 1269	SlPub24SlPub24	SlPub24SlPub24	Slpub24Slpub24	rx4rx4	22.2a
LA 2181	Slpub24Slpub24	SlPub24SlPub24	SlPub24SlPub24	rx4rx4	21.2a
LA 0395	Slpub24Slpub24	SlPub24SlPub24	SlPub24SlPub24	rx4rx4	
Ha 7998	Slpub24Slpub24	SlPub24SlPub24	SlPub24SlPub24	rx4rx4	
FG16-804	Slpub24Slpub24	SlPub24SlPub24	SlPub24SlPub24	rx4rx4	
LA 2283	Slpub24Slpub24	SlPub24SlPub24	SlPub24SlPub24	Rx4Rx4	15.6ab
Fla 8233	Slpub24Slpub24	SlPub24SlPub24	SlPub24SlPub24	Rx4Rx4	
FG16-813	Slpub24Slpub24	SlPub24SlPub24	SlPub24SlPub24	Rx4Rx4	
LA 0400	Slpub24Slpub24	SlPub24SlPub24	SlPub24SlPub24	Rx4Rx4	not included
LA 2183	Slpub24Slpub24	SlPub24SlPub24	SlPub24SlPub24	Rx4Rx4	not included
PI 114490	SlPub24SlPub24	SlPub24SlPub24	SlPub24SlPub24	rx4rx4	13.3b
FG16-802	SlPub24SlPub24	SlPub24SlPub24	SlPub24SlPub24	rx4rx4	
Ha 7981	Slpub24Slpub24	Slpub24Slpub24	Slpub24Slpub24	Rx4Rx4	11.0b
TD-55C-h	Slpub24Slpub24	Slpub24Slpub24	Slpub24Slpub24	Rx4Rx4	
ZF084-1-h	Slpub24Slpub24	Slpub24Slpub24	Slpub24Slpub24	Rx4Rx4	
Black Cherry	SlPub24SlPub24	SlPub24SlPub24	SlPub24SlPub24	Rx4Rx4	9.3b
LA 0373	SlPub24SlPub24	SlPub24SlPub24	SlPub24SlPub24	Rx4Rx4	
PI 128216	SlPub24SlPub24	SlPub24SlPub24	SlPub24SlPub24	Rx4Rx4	
Nongdazhenzhufanqie	SlPub24SlPub24	SlPub24SlPub24	SlPub24SlPub24	Rx4Rx4	
LA 0722	SlPub24SlPub24	SlPub24SlPub24	SlPub24SlPub24	Rx4Rx4	not included
11C336	SlPub24SlPub24	SlPub24SlPub24	SlPub24SlPub24	Rx4Rx4	not included
11C337	SlPub24SlPub24	SlPub24SlPub24	SlPub24SlPub24	Rx4Rx4	not included

Tomato germplasms with fewer than three plants available for disease evaluation were not included in the least significant difference comparison. Means of diseased leaf area followed by the same letter are not significantly different at *P* ≤ 0.05 based on Duncan’s multiple range test

### *SlPub24* also confers resistance to races T1, T2, and T4

Previous studies indicate that the locus *QTL-11B* from the resistant line PI 114490 confers resistance to races T1–T4^7,9^. To check whether *SlPub24* was also resistant to races T1, T2, and T4, PI 114490, OH 88119, and *Slpub24* mutants and transgenic lines overexpressing *SlPub24* were subjected to disease evaluation. Bacterial populations were significantly smaller in PI 114490 and transgenic lines than in OH 88119 and *Slpub24* mutants (Fig. S3), suggesting that *SlPub24* also conferred resistance to races T1, T2, and T4.

### Expression of SA-related genes and SA content are affected by *SlPub24*

**Fig. 6:**
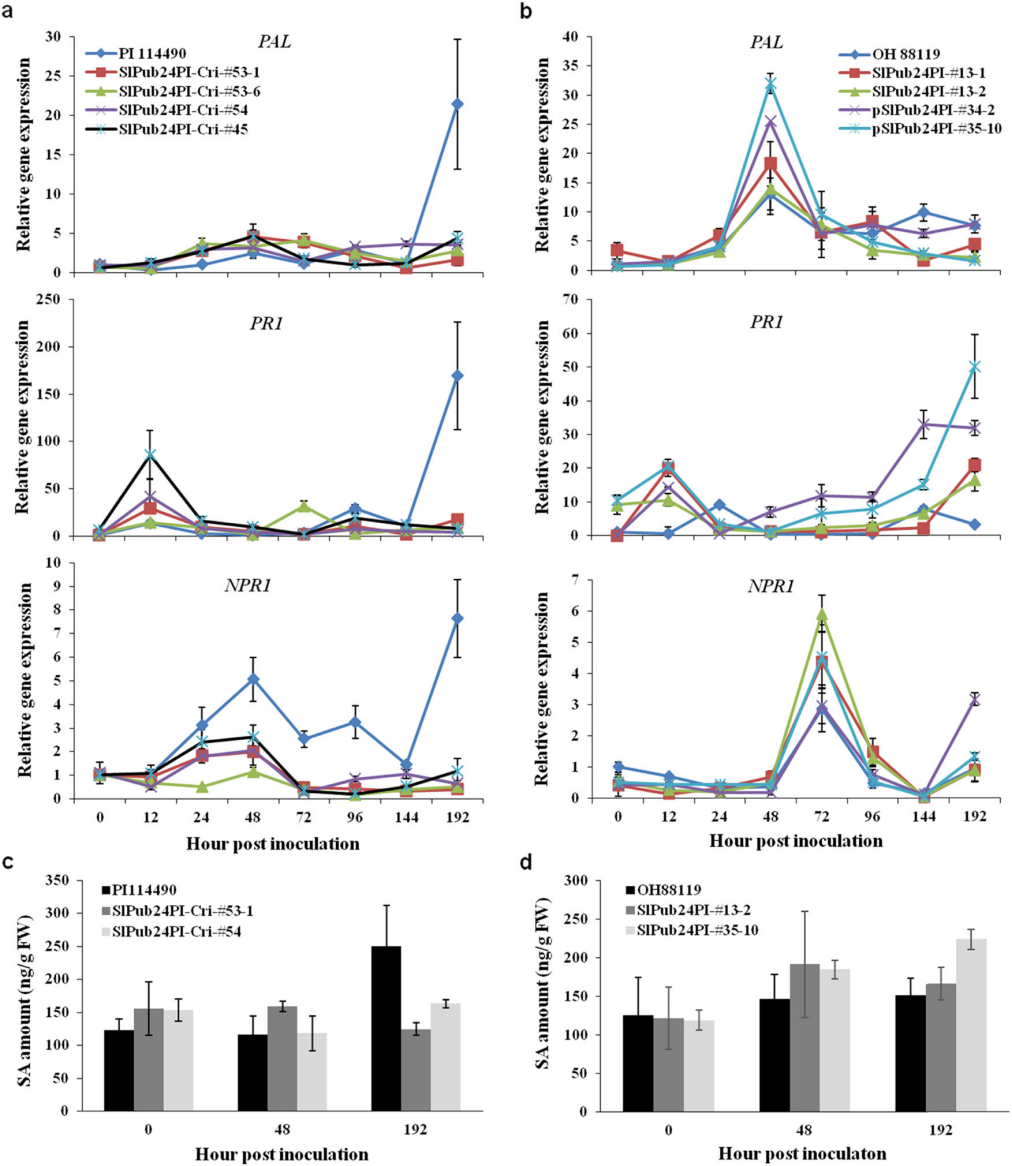
Relative expression of salicylic acid (SA)-related genes and accumulation of SA in tomato lines at various time points after spray inoculation of *Xanthomonas euvesicatoria* pv. *perforans* race T3 strain *Xv829*. **a** Relative expression of *PAL*, *PR1*, and *NPR1* in the resistant tomato line PI 114490 and *SlPub24*-mutated lines. **b** Relative expression of *PAL*, *PR1*, and *NPR1* in the susceptible tomato line OH 88119 and transgenic lines overexpressing *SlPub24* isolated from PI 114490. **c** SA content in PI 114490 and *SlPub24*-mutated lines at 0, 48, and 192 h post inoculation (hpi) with *Xv829*. **d** SA content in OH 88119 and transgenic lines overexpressing *SlPub24* at 0, 48, and 192 hpi of *Xv829*. PAL phenylalanine ammonia-lyase gene, PR1 pathogenesis-related gene 1, NPR1 nonexpresser of *PR* gene 1. Error bars represent the SD (*n* = 3)

The expression of the SA synthesis-related gene phenylalanine ammonia-lyase (*PAL*) and the signaling-related genes pathogenesis-related (*PR1)* and nonexpresser of *PR* genes 1 (*NPR1*) in PI 114490, OH 88119, and transgenic plants was measured in this study. The expression levels of *PAL*, *PR1*, and *NPR1* were low from 0 to 144 hpi and then dramatically increased from 144 to 192 hpi (Fig. 6a), similar to the expression pattern of *SlPub24* in PI 114490 (Fig. 5a). However, they were expressed at very low levels in *SlPub24*-mutated lines from 0 to 192 hpi (Fig. 6a). In transgenic lines overexpressing *SlPub24* isolated from PI 114490, the expression of *PAL*, *PR1*, and *NPR1* was higher at 48, 192, and 72 hpi, respectively, than in OH 88119 (Fig. 6b). The content of SA was consistent with the expression levels of *SlPub24*, *PAL*, *PR1*, and *NPR1*. The amount of SA in PI 114490 increased at 192 hpi but remained at a constant level in *SlPub24*-mutated lines (Fig. 6c). Meanwhile, the content of SA was higher in transgenic lines overexpressing *SlPub24* than in OH 88119 at 48 and 192 hpi (Fig. 6d). These results suggested that SlPUB24 might affect SA synthesis and signaling, which eventually influence resistance to bacterial spot.

### SlPUB24 interacts with and promotes degradation of SlCWP

**Fig. 7:**
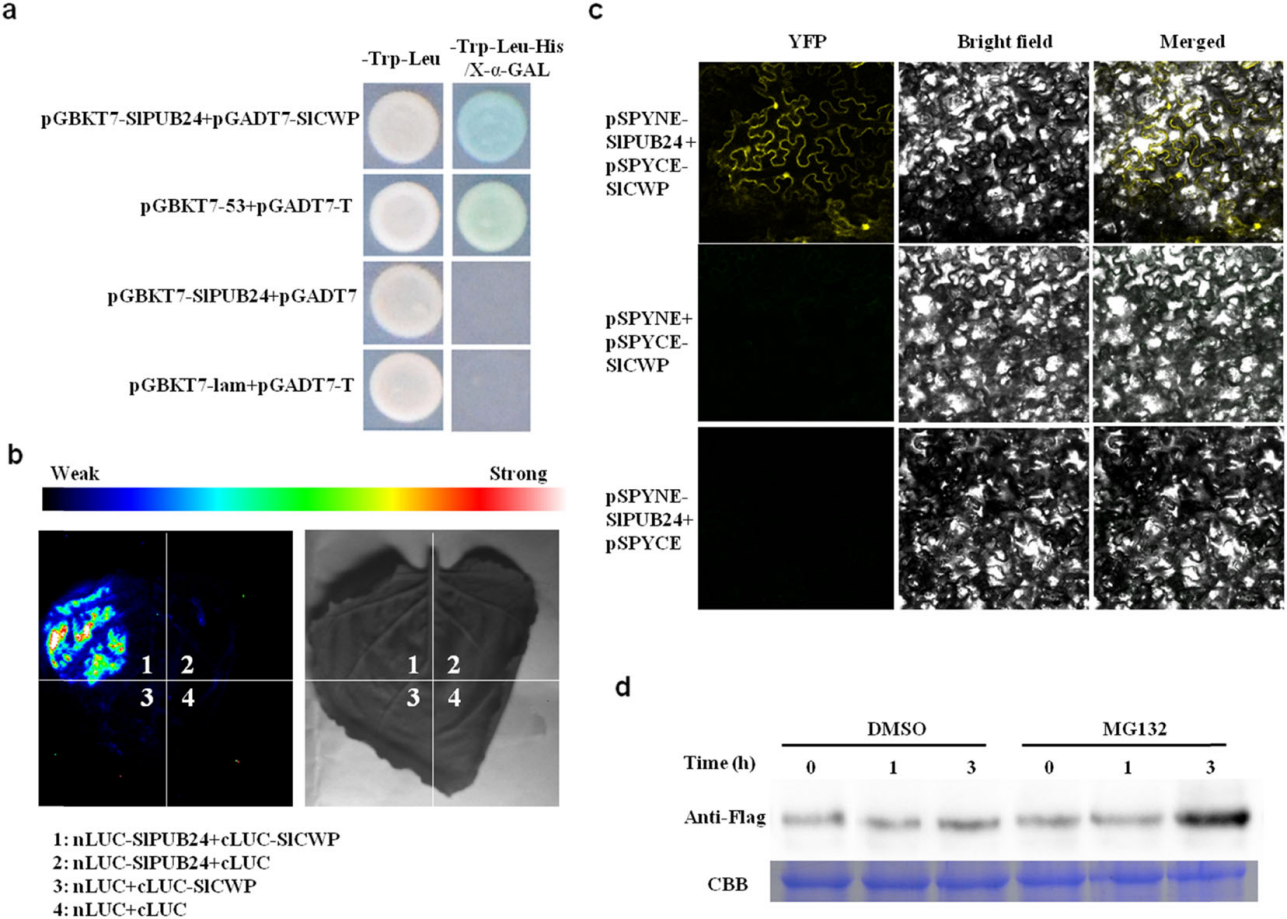
SlPUB24 interacts with SlCWP and regulates the degradation of SlCWP. **a** Yeast-two-hybrid assay. The interactions between pGADT7 and pGBKT7-SlPUB24 and between pGADT7-T and pGBKT7-lam were used as negative controls, and the interaction between pGADT7-T and pGBKT7-53 was used as a positive control. **b** Luciferase complementation image assay. Fluorescence signal intensity represents the interaction of the two proteins. **c** Bimolecular fluorescence complementation assay. YFP fluorescence was detected by confocal microscope. **d** The protein levels of SlCWP-Flag in tobacco leaves at 0, 1, and 3 h after treatment with the proteasomal inhibitor MG132 (50 mM) or an equivalent volume of dimethylsulfoxide (DMSO, control) were determined by immunoblot analysis with the Flag antibody. The concentration of total protein was monitored by Coomassie brilliant blue (CBB) staining. Molecular weight of protein: SlCWP = 20.22 kDa

To gain insight into the regulation of SlPUB24, yeast two-hybrid (Y2H) assays were performed to identify proteins that might interact with SlPUB24. After multiple screenings using deficient solid medium, 246 positive colonies associated with 55 genes were obtained, and 48 genes were finally obtained by PCR amplification. Constructs of 29 genes were successfully developed for interaction validation using the full-length CDS, and six genes (Table 2) showed interactions with SlPUB24 in three independent Y2H experiments. One gene, *Solyc02g085950* (designated *SlCWP*), represented by 21 colonies encoding the cell wall protein X77373, was selected for further investigation because it has been reported that cell wall proteins participate in disease defense^32^. The interaction between SlPUB24 and SlCWP detected by Y2H (Fig. 7a) was verified by bimolecular fluorescence complementation assay (BiFC) and split luciferase complementation assay (SLC) (Fig. 7b, c).

**Table 2:** Information on proteins interacting with SlPUB24 in tomato.

Gene ID	Function annotation
Solyc12g005630	Cytochrome b6-f complex iron-sulfur subunit
Solyc03g034220	Ribulose bisphosphate carboxylase small chain 2B
Solyc02g085950	Cell wall protein X77373
Solyc08g028690	NAD(P)-binding Rossmann-fold superfamily protein
Solyc02g094120	Sulfite oxidase
Solyc06g071050	Hypersensitive-induced response protein

A previous study showed that proteins containing the U-box domain have ubiquitin ligase E3 activity that leads to protein degradation by the 26 S proteasome. MG-132 is a cell-permeable proteasome inhibitor and can block the proteolytic activity of the 26 S proteasome complex^33^. To further prove the relationship between SlPUB24 and SlCWP, SlPUB24-Myc and SlCWP-Flag were cloned into the vector, and an *Agrobacterium*-mediated transient expression assay was implemented in *N. benthamiana* leaves. Immunoblot analysis indicated that SlCWP gradually accumulated and was not degraded by SlPUB24 in tobacco leaves treated with MG132 (Fig. 7d), which indicated that SlPUB24 might promote the degradation of SlCWP.

### The expression of *SlCWP* is opposite to that of *SlCWIINV1* during the infection of *X. euvesicatoria* pv. *perforans* race T3

**Fig. 8:**
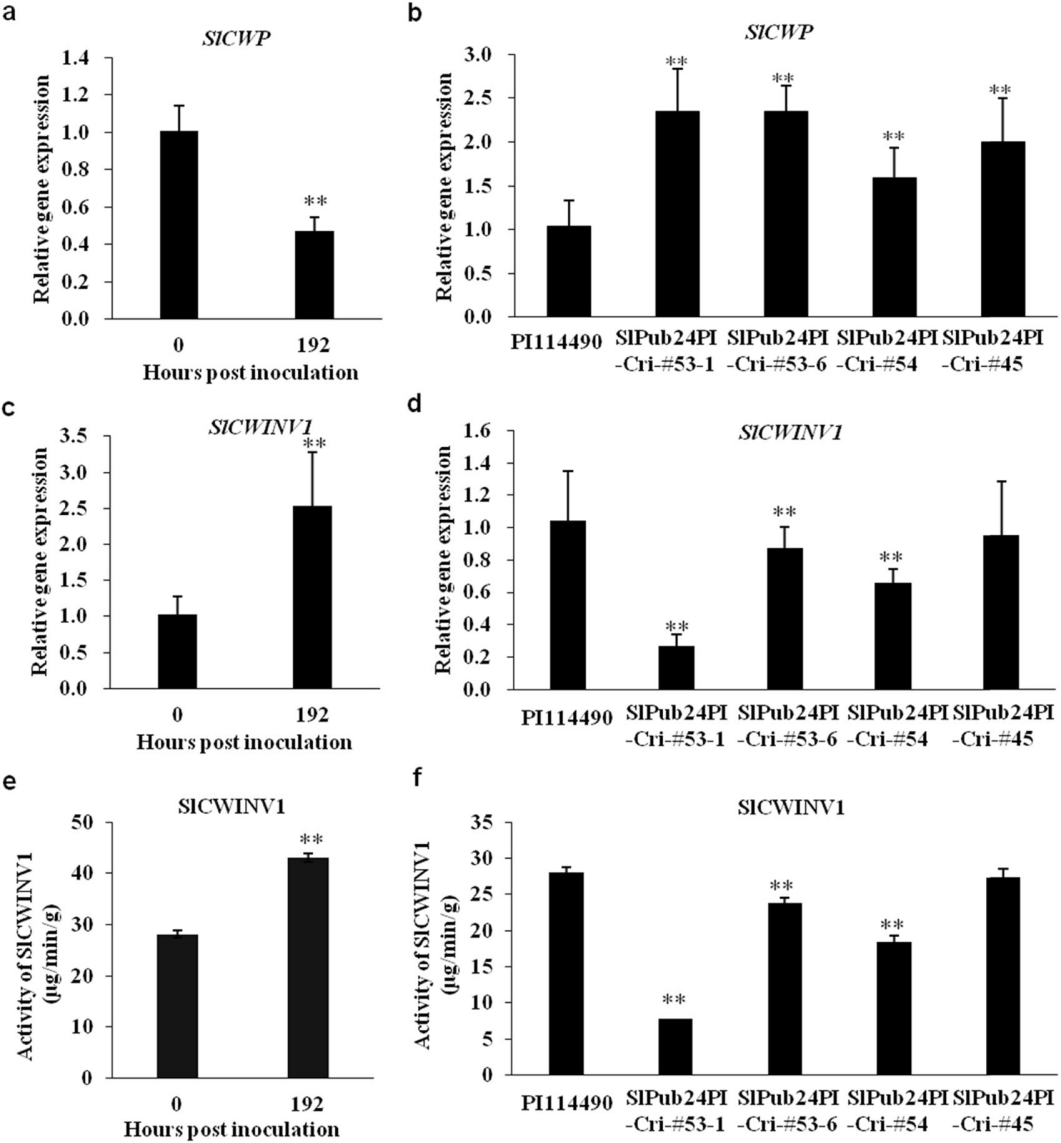
Relative expression of *SlCWP* and *SlCWINV1* as well as enzyme activity of SlCWINV1 in leaves of plants after inoculation with *Xanthomonas euvesicatoria* pv. *perforans* race T3 strain *Xv829*. **a** Relative expression of *SlCWP* in PI 114490 at 0 and 192 h post inoculation (hpi). **b** Relative expression of *SlCWP* in PI 114490 and *Slpub24* mutants generated by CRISPR/Cas9 editing at 192 hpi. **c** Relative expression of *SlCWINV1* in PI 114490 at 0 and 192 hpi. **d** Relative expression of *SlCWINV1* in PI 114490 and *Slpub24* mutants generated by CRISPR/Cas9 editing at 192 hpi. **e** Activity of SlCWINV1 in PI 114490 at 0 and 192 hpi. **f** Activity of SlCWINV1 in PI 114490 and *Slpub24* mutants generated by CRISPR/Cas9 editing at 192 hpi. Error bars represent the SD (*n* = 3)

The relative expression of *SlCWP* in PI 114490 was significantly lower at 192 hpi than at 0 hpi of *Xv829* (Fig. 8a). However, its expression was significantly upregulated at 192 hpi in CRISPR/Cas9-generated mutants of *SlPub24* (Fig. 8b). Previous studies have shown that increased invertase activity can enhance the levels of factors (cellulose, xylose, and galactose) involved in cell wall reinforcement^34^. The bacterium *Xv829* can grow in leaves of PI 114490, but no or fewer symptoms occur on the leaf surface of these plants compared to plants of the susceptible line OH 88119^12^ and bacterial propagation only happens below the epidermal cells in leaves of PI 114490^35^, suggesting that cell wall defense response might be distinct between resistant and susceptible tomato lines^35^. Therefore, the expression of the *SlCWINV1* (*Solyc03g121680*) gene encoding the cell wall invertase was examined here. The data showed that the expression of *SlCWINV1* was opposite to that of *SlCWP*. The relative expression of *SlCWINV1* in PI 114490 was significantly higher at 192 hpi than at 0 hpi for *Xv829* (Fig. 8c), while the gene was downregulated in the mutants (Fig. 8d). SlCWINV1 activity was consistent with gene expression. The activity of SlCWINV1 in PI 114490 was significantly higher at 192 hpi than at 0 hpi (Fig. 8e), whereas the activity of SlCWINV1 was significantly lower in the three mutants, SlPub24PI-Cri#53-1, SlPub24PI-Cri#53-6, and SlPub24PI-Cri#54, than in PI 114490 (Fig. 8f).

## Discussion

As a tomato source with broad-spectrum resistance to bacterial spot, PI 114490 has been used in various breeding programs to develop new lines with partial resistance to different species and races of *Xanthomonas*^8–10,36^. Several studies have shown that the common locus *QTL-11b* on chromosome 11 is responsible for the resistance to multiple species and races of *Xanthomonas*^7,9,10,12,13^. The phenotypic variation explained by the locus varies from 12.5 to 29.4% depending on the population of plants and the species of the pathogen used for genetic analyses^10,12,13^. Based on linkage map position^12^ and transcriptome data^14,15^, *SlPub24* was identified as a strong candidate gene in the locus for resistance to race T3, and *SlPub24* was further investigated in this study. Transgenic overexpression of *SlPub24* in the susceptible line OH 88119 increased resistance to race T3 (Fig. 3), while mutation of the gene in the resistant line PI 114490 using the CRISPR/Cas9 editing system decreased resistance to race T3 in mutants (Fig. 4). Furthermore, disease evaluation of mutants and transgenic lines showed that *SlPub24* also conferred resistance to races T1, T2, and T4 (Fig. S3). These results suggest that *SlPub24* is the gene for resistance to races T1–T4 in the locus *QTL-11B*.

Gene expression is largely dependent on its promoter activity. Sequence variation in the promoter region, including nucleotide substitution and insertion/deletion of certain fragments, can affect the timing and level of gene expression. Low expression of *fw2.2* in tomato plants with large fruits is due to one or more nucleotide substitutions in the promoter region of the gene^37,38^. The presence of an 11-bp InDel in the promoter region of the *SD1* gene disrupts a gibberellin-responsive *cis*-element, resulting in low expression of the gene in thin-stem tomato plants^39^. Similarly, insertion of an 11-bp fragment in the promoter region of the *Bs3* gene results in the loss of specific recognition by AvrBs3 from the pepper bacterial spot pathogen *X*. *euvesicatoria* pv. *euvesicatoria*^40,41^, while a deletion of 3 bp in the promoter region of the *Xa27* gene causes the loss of specific recognition by AvrXa27 of the rice bacterial blight pathogen *X*. *oryzae pv*. *oryzae*^42^. In the current study, substantial sequence variation was detected in the promoter regions of *SlPub24* between PI 114490 and OH 88119 (Table S2). Promoter activity analysis (Fig. 5c, d) indicated that the promoter of *SlPub24* in OH 88119 might have very low activity. Thus, the expression of the gene was low in OH 88119 regardless of whether there was pathogen infection (Fig. 5a). The results of swapping the promoter and CDS regions isolated from PI 114490 and OH 88119 indicated that the CDS of *SlPub24* from both PI 114490 and OH 88119 could contribute to resistance to race T3 (Fig. 5f), although sequence variation in the CDS of *SlPub24* existed between the two tomato lines. All these data suggested that the expression level of *SlPub24* was determined by the activity of its promoter.

Salicylic acid is an important signaling molecule that induces systemic acquired resistance and is associated with pathogen resistance in plants^43,44^. It has been shown that plants generate SA *via* the Phenylalanine Ammonia-Lyase (PAL) pathway^45–48^, and the relative expression of pathogenesis-related (*PR*) genes and nonexpresser of PR genes 1 (*NPR1*) are reliable indicators of the activity of SA signaling^49^. The expression of *PAL* influences the accumulation of pathogen-induced SA and is associated with disease resistance^50,51^. Various studies have shown that plant U-box proteins regulate disease resistance through the SA signaling pathway. Overexpression of *CMPG1-V*, which encodes a U-box E3 ubiquitin ligase in wheat, can improve broad-spectrum resistance to powdery mildew *via* increased expression of SA-responsive genes^26^. Overexpression of *OsPUB15* in rice causes increased expression of *PR* genes and enhanced resistance to blast strains in transgenic lines^52^. Knockdown of *OsPUB44* through RNAi significantly suppresses the expression of *PAL1* and decreases resistance to *Xanthomonas oryzae* pv. *oryzae* in transgenic rice lines^53^. In this study, the relative expression of the *PR1*, *PAL*, and *NPR1* genes increased in PI 114490 when plants were infected by race T3 strain *Xv829* but remained at a low level in lines with *SlPub24* mutated by CRISPR/Cas9 editing (Fig. 6a). In contrast, the transcription of these three genes was lower in OH 88119 than in the transgenic lines overexpressing *SlPub24* (Fig. 6b). It should also be noted that the increase in *PR1*, *PAL*, and *NPR1* expression (Fig. 6a) occurred later than the increase in *SlPub24* expression (Fig. 5a) in PI 114490. The SA content in tomato plants was consistent with the expression levels of *SlPub24*, *PAL*, *PR1*, and *NPR1* (Fig. 6c, d). These data suggested that SlPUB24 conferred resistance to bacterial spot by regulating the biosynthesis and signaling of SA.

**Fig. 9:**
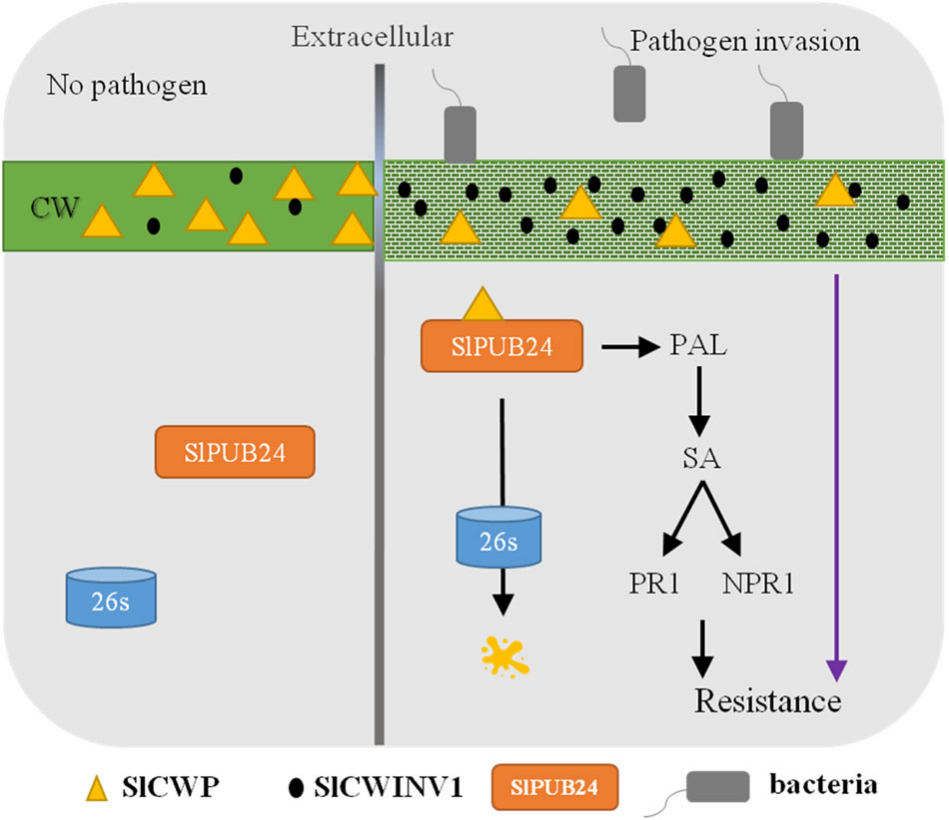
Hypothetical SlPUB24 regulatory pathway in response to pathogenic infection with bacterial spot in tomato. Plant cell walls are normal in the absence of pathogen invasion, and invertase inhibitors dynamically adjust the activity of invertase in the cell wall to stabilize the structure of the cell wall (left). When plants perceive pathogen invasion, SlPUB24 is recruited to the cell wall to interact with the cell wall protein SlCWP for degradation by the 26S proteasome. The reduction in SlCWP may increase the activity of the cell wall invertase SlCWINV1 to strengthen the cell wall for defense against pathogens. *SlPub24* also affects salicylic acid (SA) synthesis by regulating the expression of *PAL*, which results in subsequent alteration of *PR1* and *NPR1* expression. Through the SA pathway, signals are transmitted to downstream defense-related genes and activate the systemic resistance response

The plant cell wall is the first barrier to pathogen infection, as it can prevent pathogens from entering the cells. It is also the matrix for many proteins involved in pathogen perception. By destroying the cell wall, the pathogen exposes the cell to itself, causing a series of innate immune reactions in plants^32^. Cell wall invertase (CWI) responds to wounding and pathogen infections. Elevated CWI activity induces resistance to *Pseudomonas syringae* pv. *tomato* DC3000 in melatonin-treated *Arabidopsis*^34^. CWI can be regulated by specific invertase inhibitor proteins, such as cell wall/vacuolar invertase inhibitors (C/VIFs). AtC/VIF1 showed specific inhibition of VI activity, but AtC/VIF2 inhibited CWI and VI^54^. A previous study showed that bacteria can enter and propagate in the leaves of PI 114490 plants but are restricted to spongy cell layers due to the formation of wall appositions at the junction between adjacent mesophyll cells^35^. Thus, the cell wall of PI 114490 might function to prohibit bacterial migration. In this study, the interaction of the cell wall protein SlCWP with SlPUB24 was identified through Y2H and verified by BiFC and SLC (Fig. 7). The expression of *SlCWP* and *SlCWINV1* showed the opposite patterns (Fig. 8), suggesting that *SlCWP* might inhibit the expression of *SlCWINV1* during pathogen infection. However, the expression of *SlCWP* showed the same pattern as *SlPub24*. Therefore, it was most likely that SlPUB24 recruited and degraded SlCWP during pathogen infection, removing the inhibition of SlCWP on SlCWINV1. SlCWINV1 plays a role in cell wall reinforcement to form wall appositions to prevent bacterial migration (Fig. 9). Meanwhile, *SlPub24* affected the expression of *PAL*, resulting in changes in SA content and subsequently influencing the expression of *PR1* and *NPR1*, which eventually activated plant resistance.

## Materials and methods

### Plant materials

*Solanum lycopersicum* var. *cerasiforme* accession PI 114490 with field resistance^8,12^ and *S*. *lycopersicum* variety OH 88119 without resistance to *Xanthomonas euvesicatoria* pv. *perforans* race T3^4,6^ were used for gene isolation, gene expression, and genetic transformation. *Nicotiana benthamiana* plants were used for *Agrobacterium*-mediated transient expression analysis. A germplasm collection consisting of 192 tomato lines^31^ and three near-isogenic lines (NILs), FG16-802 carrying the *SlPub24* gene from PI 114490, FG16-804 carrying a QTL on chromosome 11 from Hawaii 7998, and FG18-813 carrying the QTL from Hawaii 7998 and the *Rx4* gene from PI 128216, was subjected to genotyping with the marker for selection of the *SlPub24* gene, and 26 (Table 1) were selected for disease evaluation. The NILs with partial field resistance to race T3 were kindly provided by Dr. David M. Francis at The Ohio State University. All plants were grown in greenhouses with water and fertilizer supplied unless otherwise described.

### Molecular cloning and bioinformatic analysis of *SlPub24* and *SlCWP*

Based on previous map position^12^ and transcriptome data^14^, *SlPub24* was selected as a candidate gene for the locus *QTL-11B* on chromosome 11 conferring resistance to race T3. The full-length cDNAs and promoters were amplified from PI 114490 and OH 88119 using gene-specific primers (Table S1). The open reading frame was predicted using ORFfinder in NCBI (https://www.ncbi.nlm.nih.gov/orffinder/). Alignment of deduced amino acid sequences between PI 114490 and OH 88119 was performed using Clustal X (http://www.clustal.org/). Functional domains were predicted using the SMART online tool (http://smart.embl-heidelberg.de/), and phylogenetic trees were created by MEGA X^55^ using the neighbor-joining method with 1000 bootstrap replicates. Bootstrap values are shown as percentages.

### RNA isolation and quantitative RT-PCR analysis

Total RNA was isolated from tomato leaves using the Quick RNA Isolation Kit (Huayueyang Biotechnology Co., Beijing, China) following the manufacturer’s instructions. The concentration of total RNA was determined using a NanoDrop 2000 Spectrophotometer (Thermo Fisher Scientific, Delaware, USA). Single-stranded cDNA was synthesized using the Prime Script™ RT reagent Kit with gDNA Eraser (Takara Bio Inc., Dalian, China) following the manufacturer’s instructions. Quantitative RT-PCR was performed in a 10-μl reaction volume containing 1 μl of diluted cDNA, 5 μl TB Green™ Premix Ex Taq™ (Tli RNaseH Plus) (TaKaRa), 0.25 μl ROX Reference Dye (50×), 0.25 μl each of forward and reverse gene-specific primers (Table S1), and 3.25 μl sterile purified water. The tomato *EF1-ɑ* (*Solyc06g005060*) or *actin* (*Solyc11g005330*) gene was used as an internal reference gene^15,56^. Relative expression values were determined using the comparative Ct method (2^-ΔΔCt^)^57^.

### Determination of *SlPub24* promoter activity

Measurement of promoter activity was performed by GUS assay. Promoters of the *SlPub24* gene amplified from genomic DNA of PI 114490 and OH 88119 were separately fused with the GUS reporter and cloned into the pCAMBIA1305.1 vector. The resulting constructs were transiently expressed in *N. benthamiana* leaves using the *Agrobacterium*-mediated transfection method. *A*. *tumefaciens* GV3101 and pCaMV35S::GUS were used as negative and positive controls, respectively. The infiltrated leaves were harvested 3 days after infiltration. Leaf discs (5 mm in diameter) were histochemically stained with 5-bromo-4-chloro-3-indolyl b-D-glucuronide (X-Gluc) for 24 h at 37 °C and then incubated in 70% ethanol for 48 h to remove chlorophyll before photographing. For measurements of GUS activity, 4-methylumbelliferyl beta-D-glucuronide (4-MUG, Sigma-Aldrich, USA) was added as a substrate for the fluorometric assay using the method previously described^58^, and the 4-MU produced in the GUS reaction was measured by a Thermo Scientific Microplate Reader (Thermo Fisher Scientific, DE, USA). The concentration of total protein extracted from the leaf discs was measured using a Micro BCA Protein Assay Kit (CoWin Biotech Co. Ltd., Jiangsu, China). Final GUS activity was calculated according to the standard curve of 4-MU (Sigma Aldrich) and expressed as nmol.4-MU mg^−1^ protein min^−1^.

To compare the promoter activities of the *SlPub24* gene during bacterial infection in PI 114490 and OH 88119, the expression of the GUS reporter driven by the 2.4 kb promoter isolated from PI 114490 in OH 88119 plants and driven by the 2.4 kb promoter isolated from OH 88119 in PI 114490 plants was measured at 0, 0.25, 0.5, 1, 2, 6, 12, and 24 h post inoculation (hpi) of *X. euvesicatoria* pv. *perforans* race T3 strain *Xv829*. The promoter of CaMV35S fused with the GUS reporter was cloned into pCAMBIA1305.1 and used as the positive control, while *A. tumefaciens* GV3101 was used as the negative control. The specific primers for amplification of promoters are listed in Table S1.

### Overexpression of *SlPub24* in the susceptible line OH 88119

Overexpression of *SlPub24* isolated from the resistant line PI 114490 in the susceptible line OH 88119 was performed to determine the role of *SlPub24* in resistance to race T3. Two constructs were created for genetic transformation. The first construct, SlPub24PI (Fig. S2a), was developed by inserting the fragment of the coding sequence (CDS) of *SlPub24* with a His tag into the vector pBI121^59^. The CDS fragment was amplified from the cDNA of PI 114490 using gene-specific primers (Table S1). The second construct, pSlPub24PI (Fig. S2b), was generated by inserting a fragment of 3831 bp including the promoter, 5′UTR, CDS, and 3′UTR of the *SlPub24* gene into pBI121 with excision of the CaMV 35 S promoter. The DNA fragment was amplified from the genomic DNA of PI 114490 using a pair of specific primers (Table S1). Both destination constructs were confirmed by sequencing, separately transformed into *A. tumefaciens* strain C58 using electroporation, and then separately transformed into the susceptible tomato line OH 88119 using previously described methods^60^ with slight modifications. The transgenic tomato lines were verified by PCR using primers (Table S1) specific to each construct.

### Mutation of the *SlPub24* gene in the resistant line PI 114490 using the CRISPR/Cas9 editing system

The CRISPR/Cas9 vector^61^ with modification by replacing the *Arabidopsis U6* gene promoter with the tomato *U6* gene promoter was kindly provided by Dr. Xia Cui at the Institute of Vegetables and Flowers at the Chinese Academy of Agricultural Sciences (Beijing, China). Two target sites (sgRNA1 and sgRNA2) of 20 nucleotides in the U-box domain separated by 57 bp were selected using CRISPR-P (http://cbi.hzau.edu.cn/cgi-bin/CRISPR). The CRISPR/Cas9 construct was generated following a previous description^61^. The vectors were introduced into *A. tumefaciens* strain C58 through electroporation and transformed into tomato line PI 114490 using the methods described above. All regenerated T0 lines were subjected to Cas9 detection by PCR using specific primers (Table S1), and only lines containing Cas9 were retained for further detection of mutations in the *SlPub24* gene region by sequencing PCR products amplified using a forward primer to the left of sgRNA1 and a reverse primer to the right of sgRNA2 (Table S1). Only homozygous mutants from the T2 generation were used for disease evaluation.

### Subcellular localization

The open reading frame of *SlPub24* without the termination codon was inserted into the modified pSuper1300 plasmid containing GFP protein at the *Xba* I and *Kpn* I (New England BioLabs, MA, USA) sites to generate the vector. The construct was transformed into *A. tumefaciens* by heat shock and into tomato protoplast and onion epidermal cells by PEG and gene gun, respectively. DAPI staining solution (Huayueyang Biotechnology Co., Beijing, China) was added to the transfected protoplasts for 5–10 min, followed by washing with buffer solution 2–3 times. GFP fluorescence was monitored by excitation at 488 nm, and the DAPI-stained nuclei were observed by excitation at 360 nm with an argon laser using an Olympus BX 51 fluorescence microscope (Olympus Corporation, Tokyo, Japan).

### Disease evaluation

*X. euvesicatoria* pv. *euvesicatoria* (race T1) strain *Xcv110c*, *X*. *vesicatoria* (race T2) strain *Xv1111*, *X. euvesicatoria* pv. *perforans* race T3 strain *Xv829*, and *X*. *euvesicatoria* pv. *perforans* race T4 strain *scott1* were kindly provided by Dr. Jeffery Jones at the University of Florida. Inoculum preparation and inoculation were performed according to our previous methods^11^. Both percent diseased leaf area and bacterial population were adopted as parameters for evaluating plant resistance. The percent diseased leaf area was measured using the leaf-by-leaf approach with image analysis software ASSESS V2.0^11^ 7 days post inoculation (dpi). Bacterial populations in inoculated leaves of the tomato lines were determined by the dilution plate method^11^ at 9 dpi. Three inoculated 0.2 cm^2^ leaf discs from each plant and at least 30 plants for each genotype were sampled to monitor the bacterial population. The data were analyzed using SPSS software (IBM SPSS Statistics, version 20, New York, USA) with one-way analysis of variance (ANOVA) followed by Duncan’s test for multiple comparisons. Probability values less than 5% (*P* < 0.05) were considered significant.

### Cell death detection

Trypan blue staining was performed to visualize cell death in the leaves of the tomato plants after inoculation with *Xv829*. The inoculated leaves were boiled in trypan blue staining solution (10 ml lactic acid, 10 ml glycerol, 10 g phenol, and 10 mg trypan blue dissolved in 10 ml ddH_2_O) for 3 min and stained overnight in chloral hydrate solution (2.5 g chloral hydrate dissolved in 1 ml ddH_2_O). The stained plant leaves were mounted in 70% glycerol for observation. Photographs were taken by a Nikon D3000 Digital SLR camera (Nikon, Tokyo, Japan).

### Measurement of SlCWINV1 activity and salicylic acid content

Leaf samples were collected from three plants of each genotype at 0 and 192 h post inoculation with T3 strain *Xv829*. For the measurement of SlCWINV1 activity, leaf tissues were ground in liquid nitrogen and homogenized in 2 ml/g extraction buffer (50 mM citric acid, 250 mM sorbitol, 10 mM MgCl_2_, 10 mM KCl, and 1 mM PMSF, pH 6.0). After centrifugation (8500 *g*, 10 min, 4 °C), the pellets were washed once (10 min) with extraction buffer containing 1% Triton X-100 and twice with extraction buffer only. The cell wall pellets were resuspended in 1 ml/g assay buffer (20 mM triethanolamine, 7 mM citric acid, and 1 mM PMSF, pH 4.6) and used for the determination of SlCWINV1 activity. The activity was monitored by mixing 20–100 μl of invertase preparation, 100 μl of sucrose (100 mM in assay buffer), and the assay buffer up to a volume of 300 μl. After incubation at 37 °C for 30 min, invertase activity was measured by enzymatic determination of the released glucose in a coupled enzymatic-optical assay with hexokinase and glucose-6-phosphate dehydrogenase, according to the Jansen method^62^. Determination of salicylic acid content was performed by Jiaxing Metware Metabolic Biotechnology Company (Zhejiang, China).

### Identification of proteins that interact with SlPUB24 using yeast two-hybrid assay

The yeast two-hybrid (Y2H) assay was performed following the instructions in the Matchmaker GAL4 Two-Hybrid System & Libraries User Manual (Clontech Laboratories, Inc., CA, USA). The full-length *SlPub24* open reading frame was amplified from cDNA of PI 114490 using gene-specific primers (Table S1) and cloned into the pGBKT7 bait vector^63^. The bait vector pGBKT7-SlPUB24 and the pGADT7 prey vector (cDNA library) were cotransformed into the yeast (*Saccharomyces cerevisiae*) strain AH109 by the PEG/LiOAc method. Self-activation of each protein was inhibited by 3-amino-1,2,4-triazole (3-AT) at different concentrations depending on the gene. The cotransformed yeast strains were first grown on selective medium lacking Leu and Trp (SD/-Leu-Trp) and then transferred to SD/-Trp-Leu-His/X-α-GAL. Protein interactions were determined by the appearance of blue color 3–5 days after incubation at 30 °C.

Plasmid DNA of positive colonies was isolated using a Yeast High-Purity Plasmid Extraction Kit (Aidlab Biotechnologies, Beijing, China). The cDNA inserts were amplified (primers shown in Table S1), and the resulting PCR products were sequenced. The obtained sequences were blasted to the tomato genome sequence database in NCBI (https://www.ncbi.nlm.nih.gov/) and SGN (https://solgenomics.net/) to verify the genes. The full-length sequence of each gene was obtained from SGN and used for gene-specific primer design. Candidate genes were amplified from the cDNA of PI 114490 using gene-specific primers (Table S1), and the yeast two-hybrid method described above was used to verify their interactions with SlPUB24.

### Bimolecular fluorescence complementation (BiFC) assay

The ORFs of *SlPub24* and *SlCWP* amplified from cDNA of PI 114490 without stop codons were separately cloned into the pSY-NE and pSY-CE vectors using the Seamless Assembly Cloning Kit (Takara). The fusion vectors were transferred into *A. tumefaciens* strain GV3101 using heat-shock transformation. Then, strain GV3101 containing fusion proteins was incubated at 28 °C for 12–18 h and resuspended in infiltration buffer (0.1 mM acetosyringone, 10 mM MgCl_2_, and 10 mM MES) at concentrations of 0.8–1.2 (OD_600_). Bacteria carrying the pSY-NE construct were mixed with bacteria carrying the pSY-CE construct as well as GV3101 carrying P19 at a 1:1:1 (v/v) ratio and coinfiltrated into the leaves of six-week-old seedlings of *N. benthamiana*. The yellow fluorescent protein (YFP) signal was detected using an Olympus BX 51 fluorescence microscope (Olympus Corporation) 2 days after infiltration.

### Split luciferase complementation (SLC) assay

The SLC assay was performed as previously described^64^. Constructs of SlCWP-cLUC and SlPUB24-nLUC were cotransformed into *N. benthamiana* leaves and expressed for 48 h. The abaxial sides of leaves were sprayed with 1 mM beetle luciferin (Promega, WI, USA), and the signal was captured using a Photek camera (HRPCS5, Photek, UK).

### Protein degradation assay with MG132 treatment

The plasmids p1300SlPUB24-Myc and p1300SlCWP-Flag were coexpressed with the P19 plasmid by agroinfiltration in the leaves of *N. benthamiana*. MG132 (50 μM) was infiltrated into the leaf tissue before harvest, and DMSO was used as the control. Tissues were harvested 2 days after infiltration for protein extraction using the Plant Protein Extraction Kit (CWBIO, Beijing, China) following the manufacturer’s instructions. Western blotting was performed as described previously^65^. Anti-Flag and anti-Myc (CWBIO) were diluted 1:2000. The total protein concentration was monitored by Coomassie brilliant blue (CBB) staining.

### Statistical analysis

All samples were analyzed in triplicate, and the data are expressed as the mean ± standard deviation unless noted otherwise. Statistical significance was determined using Student’s *t* test at the 0.05 (*) and 0.01 (**) levels. All experiments were conducted at least twice with three biological replicates each time.

## Supplementary information

Supplemental documents
