# Supplementary material for: Tomato SlPUB24 enhances resistance to Xanthomonas euvesicatoria pv. perforans race T3
Source: Hortic Res. 2021 Feb 1;8:30. doi: 10.1038/s41438-021-00468-4 (PMC7848003; doi:10.1038/s41438-021-00468-4)
Supplement: Supplementary file 1 — Supplemental documents [file 41438_2021_468_MOESM1_ESM.docx]

Supplementary information for

**Tomato SlPUB24 enhances the resistance to *Xanthomonas euvesicatoria* pv. *perforans* race T3**

Xin Liu^1,2^, Ge Meng^1,2^, Mengrui Wang^1,2^, Zilin Qian^1,2^, Yaxian Zhang^1,2^ and Wencai Yang^1,2*^

***Supplementary Figures***

**Fig. S1** Alignment of coding sequences of *SlPub24* (*Solyc11g068940*) and three annotated *Pub24*-like genes (*Solyc01g007050*, *Solyc01g009320*, and *Solyc06g074140*) in tomato. * indicates consensus nucleotide.

**Fig. S2** Schematic diagram of the vector construct. **a** construct of SlPUB24PI. The coding sequence (CDS) of *SlPub24* isolated from PI114490 fused with His-tag driven by CaMV35s is cloned into vector pBI121. **b** Construct of pSlPUB24PI. UTRs and CDS of *SlPUB24* isolated from PI114490 driven by its native promoter are cloned into vector pBI121. LB: left border. NOS-T: Nos terminator. RB: right border.

**Fig. S3** Bacterial population in leaves of tomato plants at 9 days post inoculation of *X.* *euvesicatoria* pv. *euvesicatoria* (race T1) strain *Xcv110c*, *X*. *vesicatoria* (race T2) strain *Xv1111*, *X*. *euvesicatoria* pv. *perforans* race T4 strain *scott1*. **a** bacterial population in PI 114490 and mutants of *SlPub24* generated by CRISPR/Cas9 editing. **b** Bacterial population OH 88119 and transgenic lines with overexpression of *SlPub24*. Error bars represent SD (n = 30).

***Supplementary Tables***

**Table S1** **Information for primers used in this study**

**Table S2 Positions of sequence variation in promoter region of the *SlPub24* gene between tomato susceptible line OH 88119 and resistant line PI 114490**

***Supplementary References***

**Supplementary Figures**


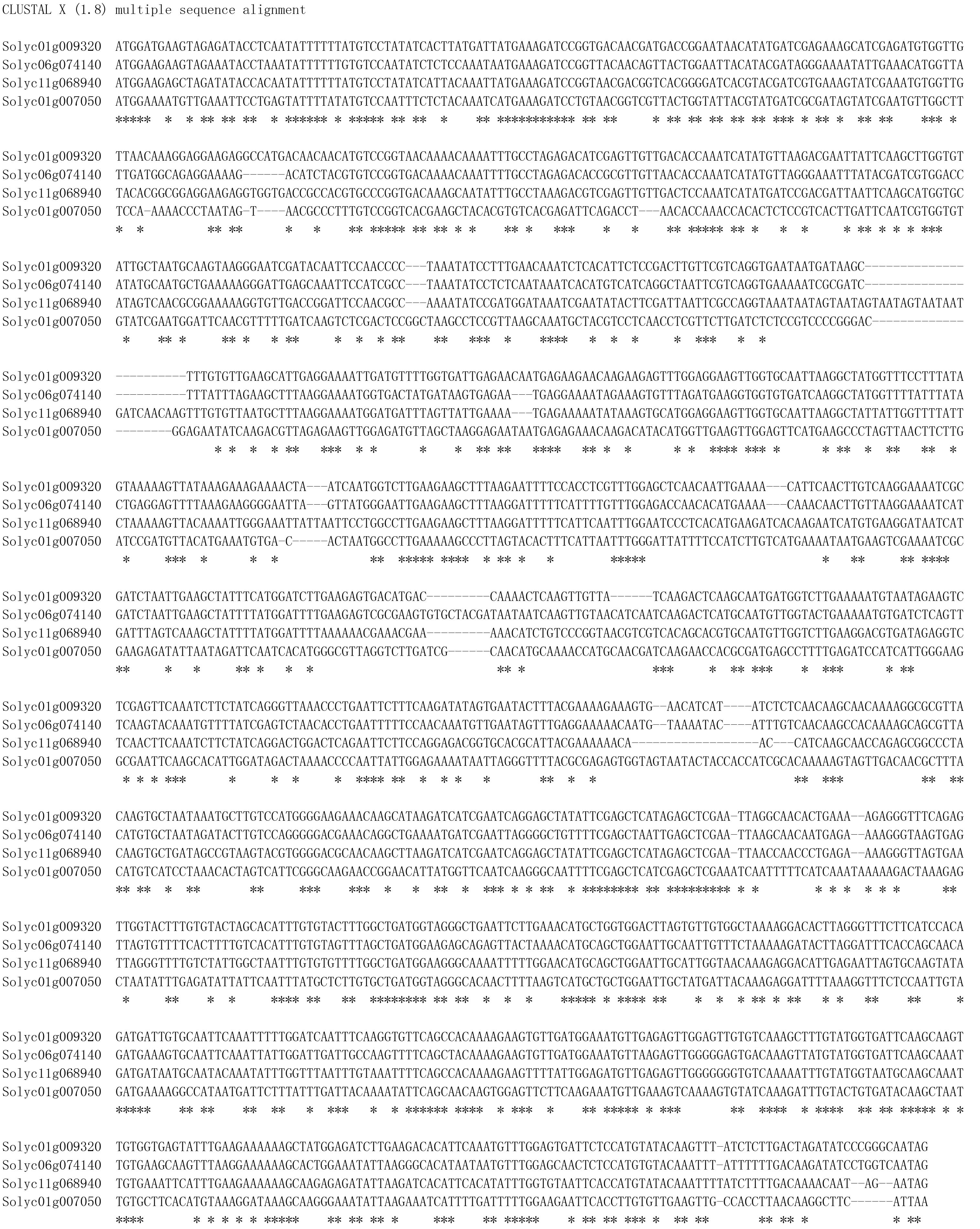


**Fig. S1** Alignment of coding sequences of *SlPub24* (*Solyc11g068940*) and three annotated *Pub24*-like genes (*Solyc01g007050*, *Solyc01g009320*, and *Solyc06g074140*) in tomato. * indicates consensus nucleotide.


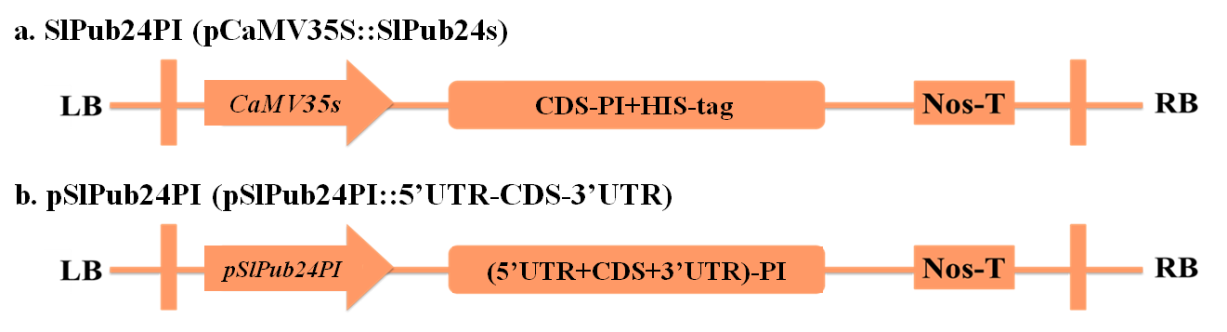


**Fig. S2** Schematic diagram of the vector construct. **a** construct of SlPub24PI. The coding sequence (CDS) of *SlPub24* isolated from PI114490 fused with His-tag driven by CaMV35s is cloned into vector pBI121. **b** Construct of pSlPub24PI. UTRs and CDS of *SlPUB24* isolated from PI114490 driven by its native promoter is cloned into vector pBI121. LB: left border. NOS-T: Nos terminator. RB: right border.


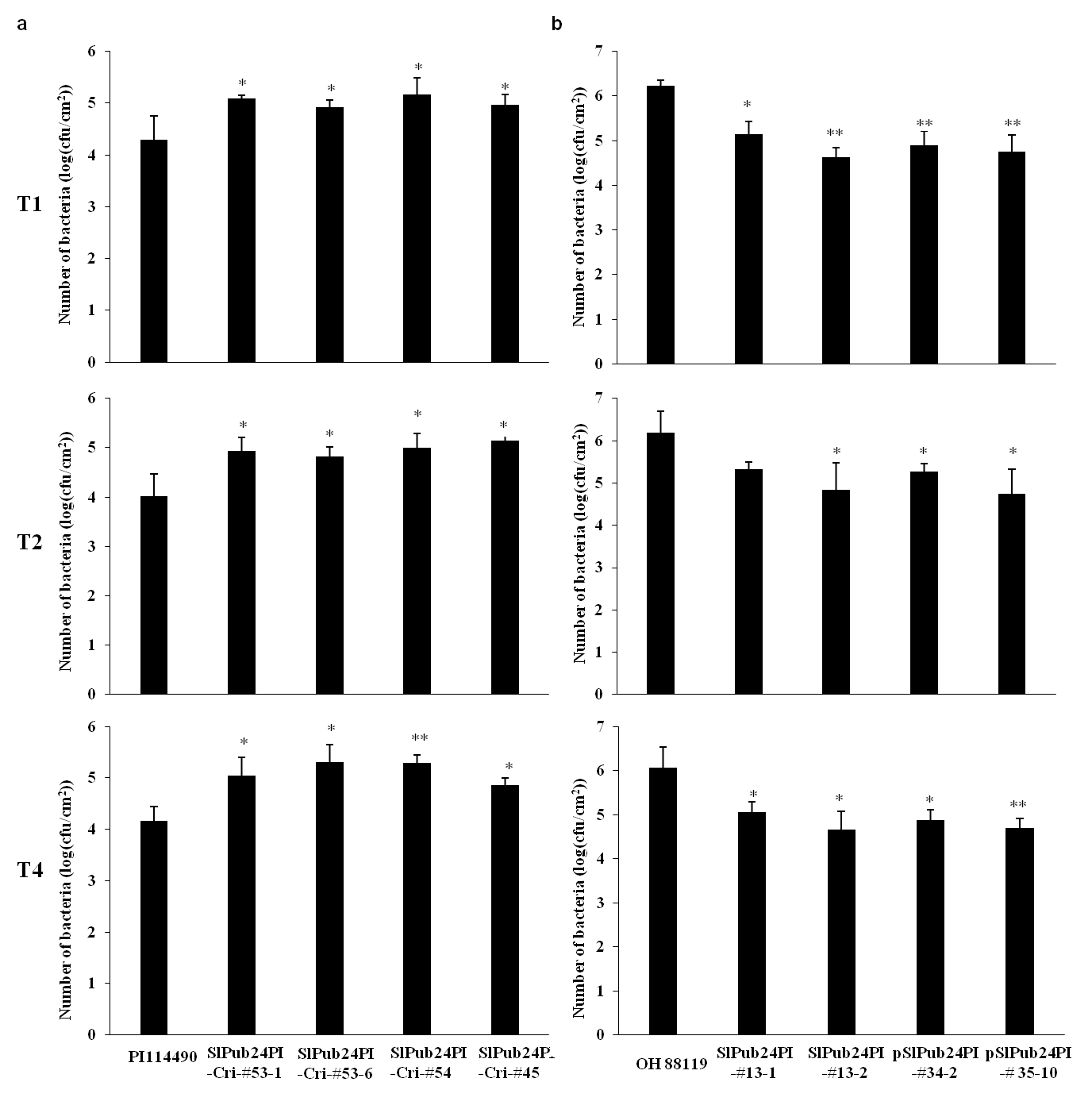


**Fig. S3** Bacterial population in leaves of tomato plants at 9 days post inoculation of *X.* *euvesicatoria* pv. *euvesicatoria* (race T1) strain *Xcv110c*, *X*. *vesicatoria* (race T2) strain *Xv1111*, *X*. *euvesicatoria* pv. *perforans* race T4 strain *scott1*. **a** bacterial population in PI 114490 and mutants of *SlPub24* generated by CRISPR/Cas9 editing. **b** Bacterial population OH 88119 and transgenic lines with overexpression of *SlPub24*. Error bars represent SD (n = 30).

**Supplementary Tables**

**Table S1 Primers used in this study**

| **Purpose** | **Primer name** | **Primer sequence (5'-3')** | **reference** |
| --- | --- | --- | --- |
| Rapid amplification of cDNA ends (RACE) | 5'RACE-GSP | GATTACGCCAAGCTTGCCGCTCTGGTTGCTTGATGGTTGTTT | this study |
|  | 3'RACE-GSP1 | GATTACGCCAAGCTTCCATCAAGCAACCAGAGCGGCCCTAC |  |
|  | 3'RACE-GSP2 | GATTACGCCAAGCTTGCCGCTCTGGTTGCTTGATGGTTGTTT |  |
| PCR amplication of *SlPub24* for subcellular localization | 1300-pub F | ACGCGTCGACATGGAAGAGCTAGATATACCACAAT | this study |
|  | 1300-pub R | GGGGTACCTTCTATTGTTTTGTCAAAAGAT |  |
| CRISPR/Cas9 editing：construct creation | Crispr-pub-F | ATATATGGTCTCGTTTGGTGGTGACCGCCACGTGCCGTTTTAGAGCTAGAAATAGC | this study |
|  | Crispr-pub-R | ATTATTGGTCTCGAAACGTGACCGTCGTTACCGGATCCAAACTACACTGTTAGATTC |  |
| CRISPR/Cas9 editing：PCR detection of Cas9 | 35SF1 | CAAGACCCTTCCTCTATATAAGGA | this study |
|  | zCas9-951R | GAGGTTATCCAGGTCATCG |  |
| CRISPR/Cas9 editing：PCR detection of target region | Cp-snp-2F | TTATGAAAGATCCGGTAACGAC | this study |
|  | Cp-snp-2R | ACGTCTTTAGGCAAATATTGCT |  |
| PCR amplification of CDS of *SlPub24* in PI 114490 for construct SlPUB24PI | pBI-His-pubF | GCTCTAGAATGGAAGAGCTAGATATACCACAATATT | this study |
|  | pBI-His-pubR | TCCCCCGGGCTAATGATGATGATGATGATGTTCTATTGTT |  |
| PCR amplification of full-length *SlPub24* in PI 114490 for construct pSlPUB24PI | PUB-all-F | GCCCTGCAGGAATTTTAAGATGATCTCACTTTGTA | this study |
|  | PUB-all-R | TCCCCCCGGGTGTGATTTCCAACTTTCTATGT |  |
| PCR Detection of transgenic lines of construct SlPUB24PI | JC-his4F | AACTTGATTCTGTCGCTACTGA | this study |
|  | JC-his4R | CTTACGGCTATCAGCACTTGTA |  |
| PCR Detection of transgenic lines of construct pSlPUB24PI | Pub-all-jCF4 | AAAGAAGTTTTATTGGAGATGTTGAG | this study |
|  | Pub-all-jCR4 | GCTTTGGACATACCATCCGTAATAAC |  |
| PCR detction of 6-bp InDel in CDS of *SlPub24*，also for detection of transgenic lines | PUBsF | CAACGCCAAAATATCCGAT | this study |
|  | PUBsR | GCCTTAATTGCACCAACTTCC |  |
| PCR amplification of promoter of *SlPub24* in PI 114490 for construct | pro-Bam2267-F | CGGGATCCAATTGCCCACGTTTGTGTCA | this study |
|  | pro-Sbf1-R | GCCCTGCAGGTTGAATGAGAAGGAAGAATGAAGAC |  |
| Yeast-two-hybrid：cDNA insertion fragment amplification | T7 | TAATACGACTCACTATAGG | this study |
|  | 3AD | AGATGGTGCACGATGCACAG |  |
| Yeast-two-hybrid：construct creation for Solyc12g005630 as AD | lx1200F | GAGGCCAGTGAATTCATGGCTTCTTCCACTCTTTCT | this study |
|  | lx1200R | GAGCTCGATGGATCCCTAAGCCCACCATGGAGC |  |
| Yeast-two-hybrid：construct creation for Solyc03g034220 as AD | lx0303F | GAGGCCAGTGAATTCATGGCTTCCTCTGTCATTTCTT | this study |
|  | lx0303R | GAGCTCGATGGATCCTTAGTAGCCTTCTGGCTTGTAG |  |
| Yeast-two-hybrid：construct creation for Solyc06g071050 as AD | lx0607F | GAGGCCAGTGAATTCATGGGCAATCTATTGGGCT | this study |
|  | lx0607R | GAGCTCGATGGATCCTCAAACTCGCCAAACATATC |  |
| Yeast-two-hybrid：construct creation for Solyc08g028690 as AD | lx0802F | GAGGCCAGTGAATTCATGCTTGAAGGTAAGGTTGC | this study |
|  | lx0802R | GAGCTCGATGGATCCCTACTTGAACATTGATAAACCTG |  |
| Yeast-two-hybrid：construct creation for Solyc02g094120 as AD | lx0209F | GAGGCCAGTGAATTCATGCCTGGGATTAAAGGG | this study |
|  | lx0209R | GAGCTCGATGGATCCCTAAAGATTTGCTTGACCAACT |  |
| Yeast-two-hybrid：construct creation for SlPUB24 as BD | BD-pub F | CCCCCCGGGAATGGAAGAGCTAGATATACCACAAT | this study |
|  | BD-pub R | ACTGCAGCTATTCTATTGTTTTGTCAAAAGAT |  |
| Yeast-two-hybrid：construct creation for SlCWP as AD | AD-cwp-F | GAGGCCAGTGAATTCATGGCTTCCTCTATAGTTTCTT | this study |
|  | AD-cwp-R | GAGCTCGATGGATCCTTAGTATCCTTCGGGCTTGT |  |
| Construct the vector of BiFC assay | CE-cwp-F | GTCCCGGGGCGGTACCATGGCTTCCTCTATAGTTTCTT | this study |
|  | CE-cwp-R | AGCTCTGCAGGTCGACTTAGTATCCTTCGGGCTTGT |  |
|  | NE-pub-F | GGACGAGCTCGGTACCATGGAAGAGCTAGATATACCACAAT |  |
|  | NE-pub-R | ACGAGATCTGGTCGACTTCTATTGTTTTGTCAAAAGAT |  |
| Construct the vector of SLC assay | nLUC-pub-F | GGACGAGCTCGGTACCATGGAAGAGCTAGATATACCACAAT | this study |
|  | nLUC-pub-R | ACGAGATCTGGTCGACTTCTATTGTTTTGTCAAAAGAT |  |
|  | cLUC-cwp-F | GTCCCGGGGCGGTACCATGGCTTCCTCTATAGTTTCTT |  |
|  | cLUC-cwp-R | AGCTCTGCAGGTCGACTTAGTATCCTTCGGGCTTGT |  |
| qRT-PCR | PAL2F | TGAAGGAATGGAATGGTGCT | SR1 |
|  | PAL2R | TGAAAGAAGCCACAAAAGTTCA |  |
|  | NPR1F | GGGAAAGATAGCAGCACG | SR2 |
|  | NPR1R | GTCCACACAAACACACACATC |  |
|  | PR-F | AACGCTCACAATGCTCGT | this study |
|  | PR-R | AAGGTCCACCAGAGTGTTGC |  |
|  | Slactin F | TGTGTTGGACTCTGGTGATGGTGT | 56 |
|  | Slactin R | ATCCAAACGAAGAATGGCATGCGG |  |
|  | SlEF1-ɑF | TACTGGTGGTTTTGAAGCTG | 15 |
|  | SlEF1-ɑR | AACTTCCTTCACGATTTCATCATA |  |
|  | SlCWI1-F | TCTCCGATCAGAGCCTTACGATTT | this study |
|  | SlCWI1-R | CCCATTCTGTAAGGTACGGGTCAG |  |
|  | CWP-F | CATGAAGAAGTACGAGACTCTG | this study |
|  | CWP-R | GTATCCTGGTGACTTATGGTTC |  |
| Construct the vector for vivo interaction | GEX-pub-F | TCCCCCCGGGGGATGGAAGAGCTAGATATACCACAAT | this study |
|  | GEX-pub-R | CCGCTCGAGCTATTCTATTGTTTTGTCAAAAGATA |  |
|  | cwp-Flg-F | ACCAAATCGACTCTAGAATGGCTTCCTCTATAGTTTCTT | this study |
|  | cwp-Flg-R | AGTATTTAAATGTCGACCGTATCCTTCGGGCTTGT |  |
|  | pub-Myc-F | ACCAAATCGACTCTAGAATGGAAGAGCTAGATATACCACAAT | this study |
|  | pub-Myc-R | AGTATTTAAATGTCGACCTTCTATTGTTTTGTCAAAAGAT |  |

**Table S2 Positions of sequence variation in promoter region of the *SlPub24* gene between tomato susceptible line OH 88119 and resistant line PI 114490**

| **Position in OH 88119** | **OH 88119** | **PI 114490** |
| --- | --- | --- |
| -141 | AA | - |
| -194 | G | A |
| -344~-347 | CGTT | - |
| -488 | A | C |
| -551~-748 | 198bp | - |
| -808 | C | T |
| -897 | A | G |
| -913 | C | A |
| -1009~-1015 | TATTTTA | - |
| -1243 | - | T |
| -1293 | - | 284bp |
| -1383 | T | - |
| -1545 | A | C |
| -1587 | A | T |
| -1716~-1718 | TTA | - |
| -1744 | T | A |
| -1861 | A | - |
| -1868 | A | T |
| -1924 | A | T |
| -1937 | C | T |
| -1969 | T | C |
| -2092 | T | C |
| -2105 | G | A |
| -2255 | G | A |
| -2279 | T | C |

**Supplementary References**

SR1 Gayoso, C., Pomar, F., Novo-Uzal, E., Merino, F. & de Ilarduya, O. M. The *Ve*-mediated resistance response of the tomato to *Verticillium dahliae* involves H_2_O_2_, peroxidase and lignins and drives *PAL* gene expression. BMC Plant Biol. **10**, 232 (2010).

SR2 El Oirdi, M. et al. *Botrytis cinerea* manipulates the antagonistic effects between immune pathways to promote disease development in tomato. Plant Cell **23**, 2405-2421 (2011).
